# In Situ Utilization of Co‐Existing Metal Ions to Fabricate Single‐Atom Catalyst for Boosting Fenton‐Like Activity

**DOI:** 10.1002/advs.202511761

**Published:** 2025-12-08

**Authors:** Jiaxing Yu, Shaohan Wang, Huajie Zhong, Zeyu Gong, Yuan Tao, Junhui Wang, Zhengping Hao, Gangfeng Ouyang

**Affiliations:** ^1^ MOE Key Laboratory of Bioinorganic and Synthetic Chemistry/KLGHEI of Environment and Energy Chemistry School of Chemistry Sun Yat‐Sen University No. 135, Xingang Xi Road Guangzhou Guangdong 510275 China; ^2^ School of Chemical Engineering and Technology Sun Yat‐Sen University Zhuhai 519087 China; ^3^ National Engineering Laboratory for VOCs Pollution Control Material & Technology University of Chinese Academy of Sciences Beijing 101408 China; ^4^ Chemistry College Center of Advanced Analysis and Gene Sequencing Zhengzhou University Zhengzhou 450001 China; ^5^ Provincial Key Laboratory of Emergency Test for Dangerous Chemicals Guangdong Provincial Engineering Research Center for Ambient Mass Spectrometry Institute of Analysis Guangdong Academy of Sciences (China National Analytical Center Guangzhou) 100 Xianlie Middle Road Guangzhou 510070 China

**Keywords:** covalent organic framework, fenton‐like reaction, in situ synthesis, single‐atom catalyst, water decontamination

## Abstract

Heavy metals and organic pollutants commonly co‐exist in the industrial wastewaters, and traditional treatment processes always treat them separately. Heavy metals can be effective active sites in heterogeneous Fenton‐like catalysts, and their potential for in situ utilization and enhancement for organic pollutant degradation is promising but still underexplored. Herein, the metal‐free TQBQ‐COF is employed with abundant pre‐designed metal‐2N anchoring sites for the in situ capture of Cu^2+^ ions for boosting organic pollutant degradation. The co‐existing Cu^2+^ can be successfully utilized to in situ fabricate a novel single‐atom catalyst (SAC) within 1 min. The coordinated Cu significantly enhances visible‐light absorption and charge separation ability, thereby substantially improving the catalyst's Fenton‐like performance (the *k* value is 240 folds higher than that in the absence of TQBQ‐COF). Singlet oxygen (^1^O_2_) and photogenerated holes (*h* ^+^) are identified as the main active species. In real wastewater with exceptionally high concentrations of Cu and chemical oxygen demand (COD), effective synergistic removal of organic pollutants and heavy metals can still be achieved, further demonstrating the applicability of this strategy. This work provides new insights into catalyst design and a new strategy of “treating waste with waste” for heterogeneous water‐treatment technologies.

## Graphical abstract

A metal‐free TQBQ‐COF with pre‐designed anchoring sites captures coexisting Cu²⁺ from wastewater to form a single‐atom catalyst within 1 min. This in‐situ fabrication boosts visible‐light photo‐Fenton activity, enabling efficient removal of organics pollutants via ^1^O_2_ and holes.

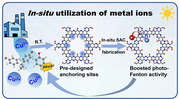

## 1 Introduction

The co‐existence of heavy metals and organic pollutants in wastewater and the environment has been a thorny problem to treat, especially in electronic manufacturing, battery waste, electroplating, etc.^[^ ^1^, ^2^, ^3^, ^4^ ^]^ Specifically, the chemical manufacturing and other sectors discharge a large volume of wastewater containing heavy metals, such as over 7.00 million tons of copper (Cu) ions per year.^[^ ^5^ ^]^ To address the co‐existing heavy metals in wastewater, traditional remediation methods rely on hydroxides (e.g., Ca(OH)_2_ or NaOH) or sulfides (e.g., FeS or H_2_S) for chemical precipitation.^[^ ^6^, ^7^, ^8^ ^]^ The chemical precipitation processes are ineffective for actual wastewater, as they only remove free metal ions (e.g., Cu^2+^) but not a significant fraction of recalcitrant metal‐organic complexes (e.g., Cu‐ethylenediaminetetraacetic acid, abbr. EDTA).^[^ ^9^ ^]^ Worse still, to subsequently remove the organic pollutants, traditional Fenton or biological processes require a large amount of acid to adjust the sewage back to neutral or acidic, resulting in additional consumption of chemical reagents.^[^ ^10^ ^]^ Moreover, residual metal ions may still hinder the degradation of organic pollutants by inhibiting the biological activity of activated sludge or poisoning the catalysis through complexation or unexpected electron transfer with the active sites and reactive species.^[^ ^11^, ^12^, ^13^ ^]^ Therefore, how to synergistically treat organic pollutants and heavy metal ions remains a significant challenge.

Heterogeneous Fenton‐like processes have been considered as promising treatment strategies for the decontamination of organic pollutants, for the advantages of mild reaction conditions, low energy and reagent consumption, and high mineralization efficiency.^[^ ^14^, ^15^, ^16^, ^17^, ^18^ ^]^ In fact, Cu can serve as a highly effective doping atom in Fenton or Fenton‐like catalysts. For example, Cu can significantly modulate the electronic structure of the catalyst or directly act as an effective active site for the activation of oxidants.^[^ ^19^, ^20^, ^21^, ^22^ ^]^ In particular, single‐atom catalysts (SACs) are considered the most effective configuration to maximize metal atom utilization efficiency.^[^ ^23^, ^24^, ^25^, ^26^, ^27^ ^]^ However, conventional ex situ metal incorporation requires complex synthesis procedures, such as high‐temperature calcination or solvent processing, and overlooks the metal ions that already exist in real wastewater. In contrast, leveraging the co‐existing Cu^2+^ ions presents substantial potential, enabling their simultaneous removal from wastewater while synergistically enhancing the organic degradation performance of Fenton‐like catalysts.

To address this research gap and explore the possibility of in situ utilization of co‐existing heavy metals, it is essential to develop an efficient and stable catalytic system with sufficient metal coordination sites. Structures such as bipyridine or phenanthroline are highly effective metal ligands, due to the strong coordination ability of the 2N structure. Previous studies have reported several SACs fabricated by anchoring metals onto the clear phenanthroline‐like bidentate 2N coordination sites on the skeleton, indicating the great potential of rapid coordination of Cu^2+^ ions from wastewater by utilizing this 2N structure.^[^ ^28^, ^29^, ^30^ ^]^ Covalent organic frameworks (COFs) have recently emerged as a promising platform for metal coordination and Fenton‐like catalysis, with distinct advantages such as well‐defined and tunable periodic structures, mild synthesis conditions, robust covalent bonding networks, and broad optical absorption capabilities.^[^ ^31^, ^32^ ^]^ Thus, COFs provide ideal scaffolds for the precise engineering of metal utilization structures.

**Scheme 1:**
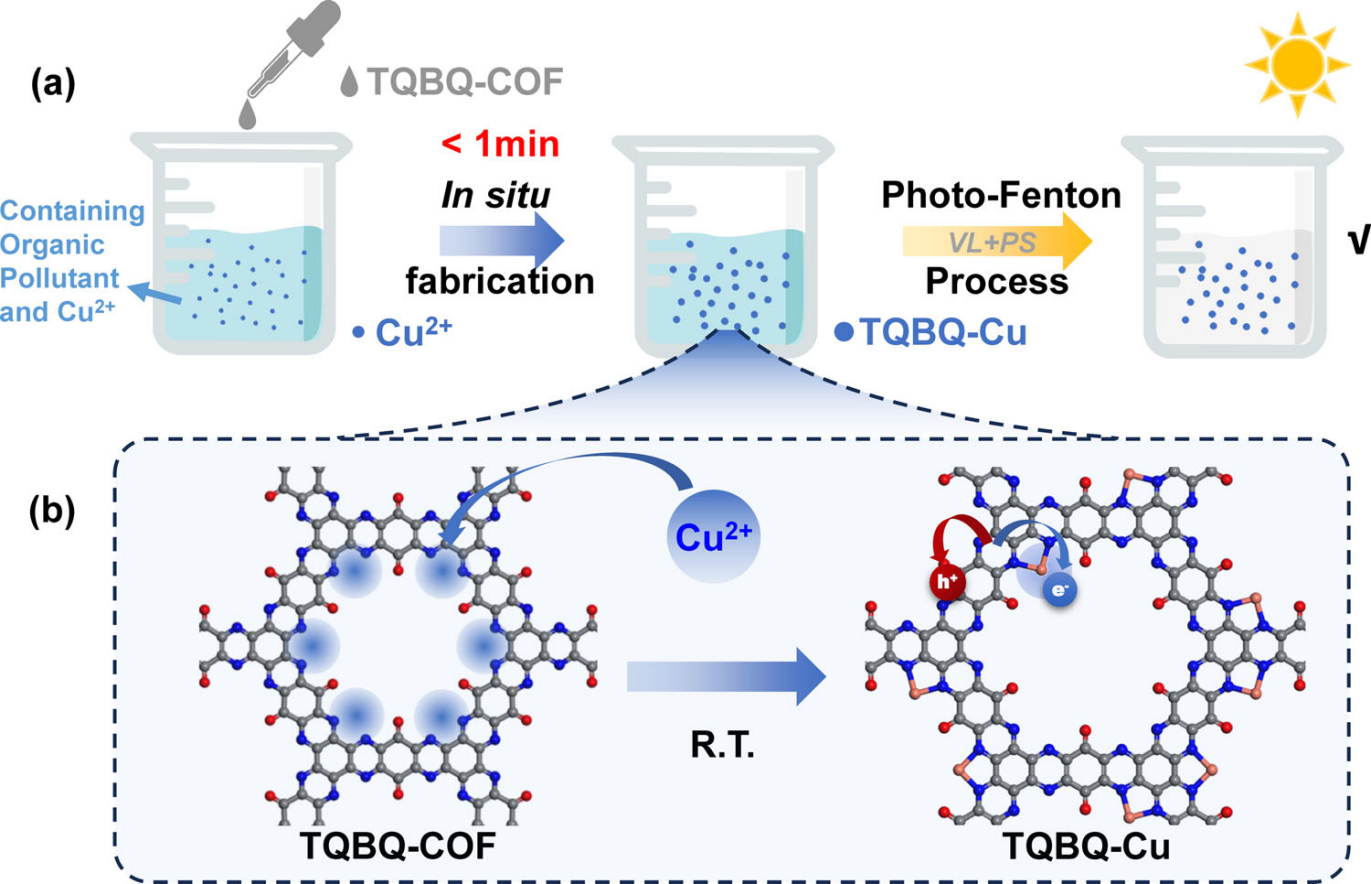
a) Schematic illustration of the in situ fabrication of TQBQ‐Cu for the photo‐Fenton process. b) Structure illustrations of TQBQ‐COF and TQBQ‐Cu.

Inspired by these, we synthesized a metal‐free COF named TQBQ‐COF (TQBQ represents triquinoxalinylene and benzoquinone units), featuring abundant pre‐designed 2N anchoring sites in its framework. It contains 21.56% N elements, indicating its ultrahigh Cu coordination ability. As expected, when Cu^2+^ ions co‐exist in the wastewater, they could be in situ captured by TQBQ‐COF within 1 min, and the photo‐Fenton activity was significantly enhanced (with 240 folds improvements than that in the absence of TQBQ‐COF) (illustrated in **Scheme** **1**). The incorporation of Cu significantly alters the electronic structure of the material, transforming the initially photo‐Fenton inactive COF into a highly active COF‐based SAC. Quenching experiments and electron paramagnetic resonance (EPR) experiments indicated that the main active species in the system were ^1^O_2_ and *h* ^+^. Notably, the TQBQ‐COF is highly effective in synergistically promoting the organic pollutant degradation and Cu removal in actual electroplating wastewater containing ultrahigh concentrations of Cu (> 4000 ppm) and chemical oxygen demand (COD) (> 20 000 ppm), demonstrating the strong applicability of this strategy for real wastewater decontamination. This work provides valuable insights into the design and facile fabrication of novel catalysts and proposes a new strategy of “turning waste into treasure” to upgrading Cu^2+^ for heterogeneous water‐treatment technologies.

## 2 Results and Discussion

### 2.1 TQBQ‐COF Synthesis

The metal‐free TQBQ‐COF was synthesized simply by polycondensation of tetramino‐benzoquinone (TABQ) and hexaketocyclohexane (HKH) (Figure S1a, Supporting Information).^[^ ^33^ ^]^ Powder X‐ray diffraction (PXRD) confirmed the high crystallinity of TQBQ‐COF, showing distinct peaks at ≈27.8°, ≈20°, and ≈18.5°, which correspond to the (002), (201), and (210) crystal planes, respectively (Figure S1b, Supporting Information).^[^ ^33^ ^]^ The diffraction peaks were in accordance with the simulated results of period DFT calculations based on a two‐layer model of AB stacking structure. The strong (002) periodic stacking lattice plane peak might be induced by the out‐of‐plane reflections of *π–π* stacking of TQBQ‐COF along the c‐axis, indicating its graphene‐like π‐conjugated 2D structure. Moreover, the chemical structure of TQBQ‐COF was further characterized by solid‐state ^13^C nuclear magnetic resonance (^13^C NMR) and Fourier transform infrared (FT‐IR) spectra (Figures S2 and S3, Supporting Information). The emerging C═N peaks at a chemical shift of 145 ppm in ^13^C NMR spectra and the adsorption group of 1520 cm^−1^ in FT‐IR spectra indicated the successful formation of C═N linkages, which was consistent with the expected structure in Figure S1a (Supporting Information).^[^ ^34^ ^]^ Moreover, the elemental compositions and elemental coordination environments were analyzed by X‐ray photoelectron spectroscopy (XPS) (Figure S4, Supporting Information). The sample has only distinct C, N, O peaks, as expected. The C 1s spectrum could be convoluted into well‐resolved components of sp^2^‐C (283.1 eV), sp^3^‐C (284.8 eV), C═N (286.4 eV), C─N (288.0 eV), and C─O (289.5 eV).^[^ ^35^, ^36^ ^]^ The N content of the synthesized TQBQ‐COF is as high as 21.56%, with clear C─N and C═N peaks in C1s and N1s spectra, implying the successful synthesis of the N‐rich COF material with pre‐designed 2N anchoring sites.

### 2.2 In Situ Catalytic Utilization of Cu^2+^ by TQBQ‐COF

**Figure 1:**
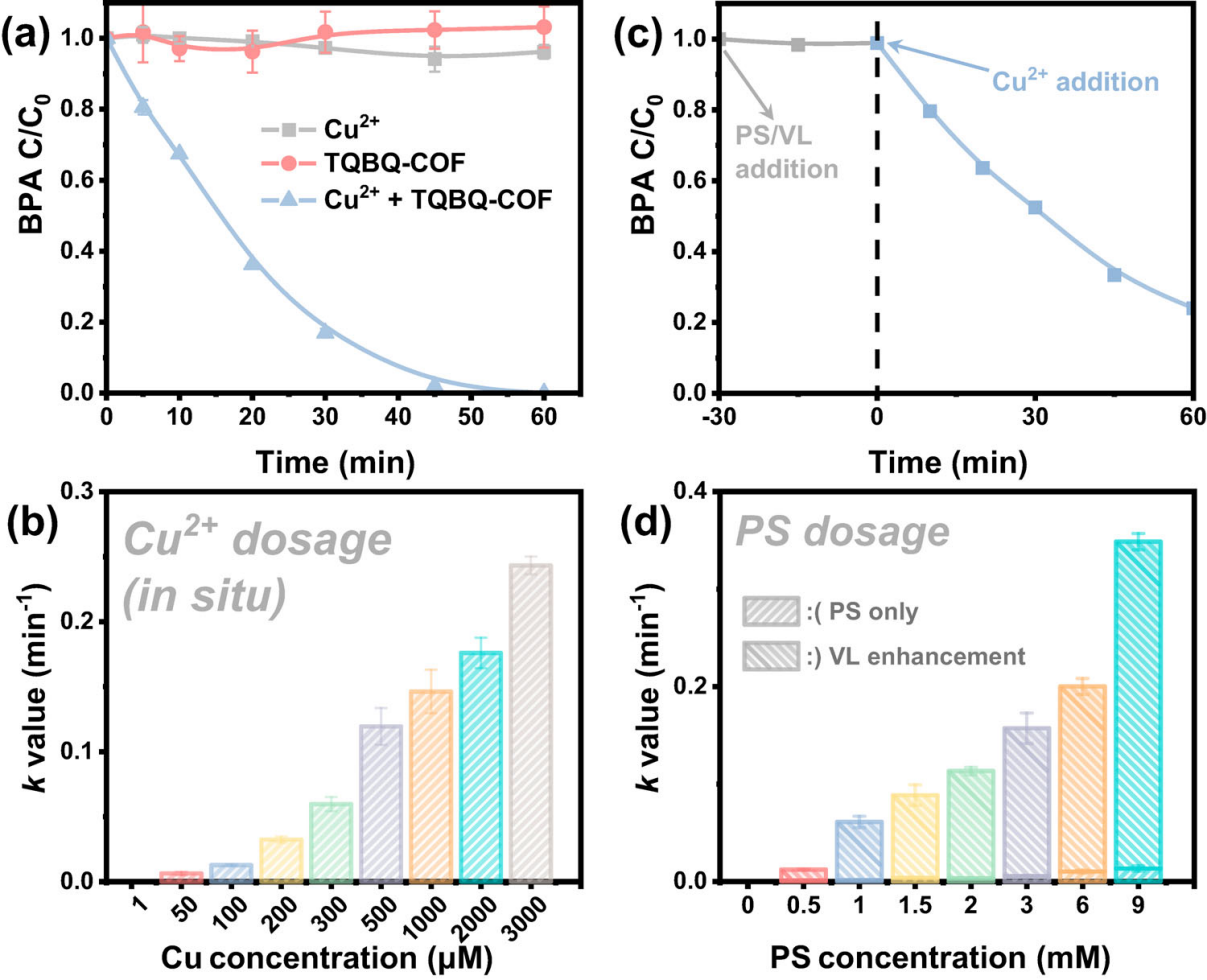
a) Catalytic activities of pure Cu^2+^, TQBQ‐COF, and TQBQ‐COF + Cu^2+^ under VL and PS addition. ([Cu^2+^] = 500 µm, pre‐adsorbed for 1 min) b) Catalytic activities of TQBQ‐COF*/*VL*/*PS system with the existence of different concentrations of Cu^2+^. c) Catalytic activities of TQBQ‐COF before and after 500 µm Cu^2+^ were instantly added under visible light and PS addition. d) The photo‐Fenton efficiency of TQBQ‐Cu (pre‐adsorbed with 500 µm Cu^2+^) under VL and different PS dosage. ([BPA] = 20 ppm, [PS] = 1.5 mm unless specified, [catalyst] = 0.1 g L^−1^).

To verify the in situ utilization capability of TQBQ‐COF toward co‐existing Cu^2+^, degradation experiments were carried out using bisphenol A (BPA) as a model organic pollutant. TQBQ‐COF alone exhibited no ability to remove BPA, either through persulfate (PS) activation or under visible light (VL) irradiation, indicating its inert nature in the absence of metal species (Figure S5, Supporting Information). However, when Cu^2+^ was pre‐adsorbed by TQBQ‐COF for 1 min, the degradation was greatly enhanced, showing the critical role and ultrafast capture of Cu^2+^ (**Figure** **1**a; Figure S6, Supporting Information). The corresponding total organic carbon (TOC) removal was also monitored and shown in Figure S7 (Supporting Information). The performances with different Cu^2+^ concentrations are shown in Figure 1b. The in situ formed TQBQ‐Cu system began to exhibit degradation activity toward BPA upon addition of a minimal 50 µm Cu^2+^, which was below one‐third of the WHO drinking water standard threshold (10 mg L^−1^). Then the photocatalytic performance increased significantly with higher concentrations of Cu^2+^, displaying a clear upward trend (Figure 1b). With the co‐existence of 3000 µm Cu^2+^, the *k* value of the TQBQ‐COF/PS/VL system reached a maximum of 0.243, which was 240 folds higher than that in the absence of TQBQ‐COF.

The Cu^2+^ coordination duration was further investigated. When Cu^2+^ was added in real time during the reaction process, the degradation was still greatly enhanced (Figure 1c). Besides, extending the pre‐adsorption time duration did not enhance the performance, further confirming the rapid Cu^2+^ adsorption (Figure S8, Supporting Information). To further prove that the co‐existing Cu^2+^ was rapidly and steadily incorporated into TQBQ‐COF, a series of TQBQ‐Cu catalysts were prepared by pre‐adding various concentrations of Cu^2+^ into an aqueous suspension of TQBQ‐COF. After 12 h stirring to allow coordination and washing to remove unbound Cu^2+^, the resulting performance was collected, as illustrated in Figures S9 and S10 (Supporting Information). Their performance trends closely matched those of the in situ fabricated TQBQ‐Cu, with the reaction rate constant (*k*) increasing with Cu^2+^ concentration until reaching a plateau at ≈1 mm. The similar performance between 1 min‐prepared in situ prepared TQBQ‐Cu and 12 h‐prepared ex situ TQBQ‐Cu confirmed the successful and rapid Cu coordination within 1 min during the in situ process. Furthermore, the Cu^2+^ leaching was monitored using ex situ fabricated TQBQ‐Cu with 0.5 mm Cu^2+^ pre‐adsorbed. The Cu^2+^ leaching concentration was measured after dispersed in pure water and after the degradation process (Figure S11, Supporting Information). The results showed that the Cu^2+^ leaching was kept far below the drinking water standard and confirmed the robust coordination of Cu^2+^.

Additionally, the influence of different PS dosage was investigated. As shown in Figure 1d and Figure S12 (Supporting Information), the photodegradation performances increased with PS dosage. Notably, the pseudo‐first *k* value increased steadily from 0.01 min^−1^ at 0.5 mm PS to 0.34 min^−1^ at 9 mm PS, indicating the excellent PS activation and VL utilization capability of TQBQ‐Cu. Moreover, comparing with other reported photocatalysts, TQBQ‐Cu/PS/VL system exhibited excellent photocatalytic performance for the degradation of BPA (Table S1, Supporting Information). The degradation of other organic compounds was also evaluated for broader research (Figure S13, Supporting Information). All model pollutants were effectively degraded by the TQBQ‐Cu/PS/VL system, indicating the versatility of the system and the in situ SAC fabrication strategy.

Overall, the rapid adsorption of Cu^2+^, remarkable catalytic enhancement, and ultrawide effective ranges of Cu^2+^ and PS concentration collectively demonstrate the flexibility and practicality of this in situ activation strategy.

### 2.3 TQBQ‐Cu Characterization and Investigation on Atomically‐Dispersed Cu Incorporation

The in situ coordination between Cu^2+^ and TQBQ‐COF is believed to be the key factor contributing to the excellent photo‐Fenton performance of the TQBQ‐Cu. Consequently, to further investigate the interaction mechanism and performance changes induced by Cu^2+^ incorporation, a series of characterization was performed for the TQBQ‐Cu. The morphology and Cu dispersion of TQBQ‐Cu were investigated using scanning electron microscopy (SEM), transmission electron microscopy (TEM), and spherical aberration corrected atomic‐resolution high‐angle annular dark‐field scanning transmission electron microscopy (AC‐HAADF‐STEM) (Figures S14–S16, Supporting Information). As shown in Figure S15 (Supporting Information), isolated Cu atoms were observed to be uniformly dispersed throughout the TQBQ‐Cu matrix, without any metallic Cu species or Cu cluster (the atomically dispersed Cu sites were visualized as bright dots, highlighted with red circles). Additionally, energy‐dispersive X‐ray spectroscopy (EDS) elemental mapping based on the dark‐field HAADF‐STEM image revealed the homogeneous spatial distributions of C, N, O, and Cu elements within the framework (Figure S16c, Supporting Information).

**Figure 2:**
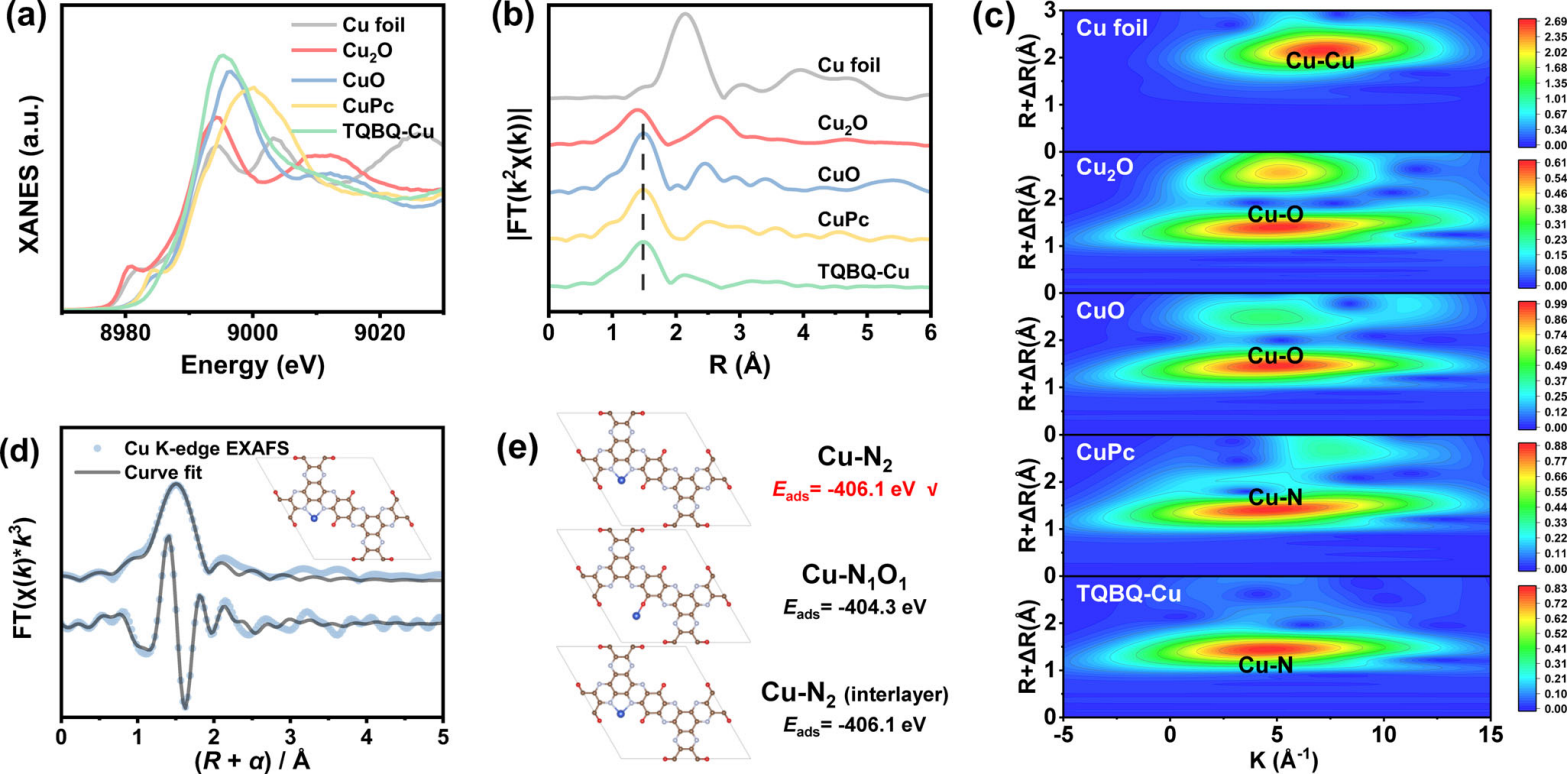
a) XANES spectra of standard substances and TQBQ‐Cu. b) EXAFS spectra of standard substances and TQBQ‐Cu. c) Wavelet transform of EXAFS spectra at Cu K‐edge. d) Cu K‐edge EXAFS (blue dot) and the curve fit (gray line) for TQBQ‐Cu, plotted in R‐space (FT magnitude and imaginary component), k^3^‐weighted. e) Calculated adsorption energy of TQBQ‐Cu at different Cu positions. The data are not phase‐corrected.

Furthermore, X‐ray absorption spectroscopy (XAS) was performed to analyze the local coordination environment of Cu atoms in TQBQ‐Cu.^[^ ^37^, ^38^ ^]^ The normalized X‐ray absorption near edge structure (XANES) spectra are presented in **Figure** **2**a. The absorption edge position of TQBQ‐Cu was located between that of CuO and Cu_2_O, indicating the average valence state of Cu species was in the range of 1–2. The Fourier‐transformed extended X‐ray absorption fine structure (EXAFS) for TQBQ‐Cu exhibited a predominant peak at 1.50 Å, corresponding to Cu─O/Cu─N coordination. Notably, no peaks associated with Cu─Cu (2.3 Å) or second‐shell Cu─O─Cu (≈2.7 Å) interactions were detected, confirming the atomically dispersed single‐atom Cu in TQBQ‐Cu (Figure 2b). Additionally, wavelet transform (WT) EXAFS at the Cu K‐edge was plotted to verify the atomic dispersion of Cu atoms (Figure 2c). A signal center at the radial distances of ≈1.5 Å (R+△R), corresponding to Cu─N coordination, was observed in both CuPc and TQBQ‐Cu, consistent with the EXAFS result.

The EXAFS spectra were then well fitted using first‐shell backscattering paths in R space (Figure 2e; Figure S17, Supporting Information). The average coordination number of Cu around N was ≈2.1. The loading sites of Cu^2+^ were subsequently explored via density functional theory (DFT) calculations (Figure 2d; Figure S18, Supporting Information). The initial position of Cu^2+^ was set at Cu‐2N, Cu‐NO, and interlayer Cu‐2N, respectively. After structural optimization, interlayer Cu‐2N was the same structure as the intralayer Cu‐2N configuration (Figure 2d). The calculations revealed that Cu^2+^ was more easily adsorbed to form the intralayer Cu‐2N coordination for TQBQ‐Cu. The overall results validated the single atomic dispersion of Cu and Cu‐2N coordination environment.

Narrow scan XPS spectra (Figure S19, Supporting Information) were adopted to further analyze the elemental coordination environments. The Cu 2p spectrum showed the coexistence of Cu (II) and Cu (I) species in TQBQ‐Cu, which was in agreement with the XANES results. Compared to TQBQ‐COF, the C═N peak significantly shrunk, which could be evidence for the formation of Cu─N covalent bond after Cu incorporation. The inference is further supported by the nearly unchanged O 1s spectra (Figure S19c,d, Supporting Information), suggesting that Cu preferentially coordinates with N rather than O atoms. FT‐IR spectra were also obtained to further determine the chemical changes of TQBQ‐COF and TQBQ‐Cu (Figure S20, Supporting Information). A significant decrease in the peak at 1550 cm^−1^ (C═N) was observed after Cu doping, while the peak at 1380 cm^−1^ (C═O) remained largely unchanged, further confirming the selective coordination of Cu with N sites.

Finally, to investigate the structural changes induced by Cu^2+^ doping, XRD, Raman spectroscopy, and thermogravimetric analysis (TGA) were conducted. After Cu doping, the XRD peaks (Figure S21, Supporting Information) of (210) crystal planes of TQBQ‐Cu were disappeared, and the (201) peak was weakened and broadened, suggesting reduced in‐plane crystallinity. In contrast, the (002) stacking peak becomes stronger and sharper, indicating enhanced crystallinity of the interlayer *π–π* stacking upon Cu^2+^ incorporation. Meanwhile, the 2θ angle position of the (002) characteristic peak downshifted from 27.8° to 27.4°, implying an increased interlayer spacing due to Cu doping.^[^ ^39^ ^]^ Raman spectroscopy (Figure S22, Supporting Information) further supported these structural changes, and the G band centered at 1520 cm^−1^ represented to the in‐plane bond‐stretching vibrations of sp^2^ carbon atoms, confirming the graphene‐like structure of TQBQ‐COF.^[^ ^40^ ^]^ The height ratio of defect‐induced band (D band) to G band (I_D_/I_G_) was 0.77 for TQBQ‐COF. A slightly lower I_D_/I_G_ value of 0.72 was observed in TQBQ‐Cu, indicating an increase in crystallinity degree, which was consistent with the XRD results.

In conclusion, the co‐existing Cu^2+^ was coordinated on the well‐defined pre‐designed 2N coordination sites as expected, and the in situ prepared TQBQ‐Cu was confirmed as a novel and robust COF‐based Cu SAC.

### 2.4 Mechanism Investigation on Single‐Atom Cu Boosting Photocatalytic Performance

**Figure 3:**
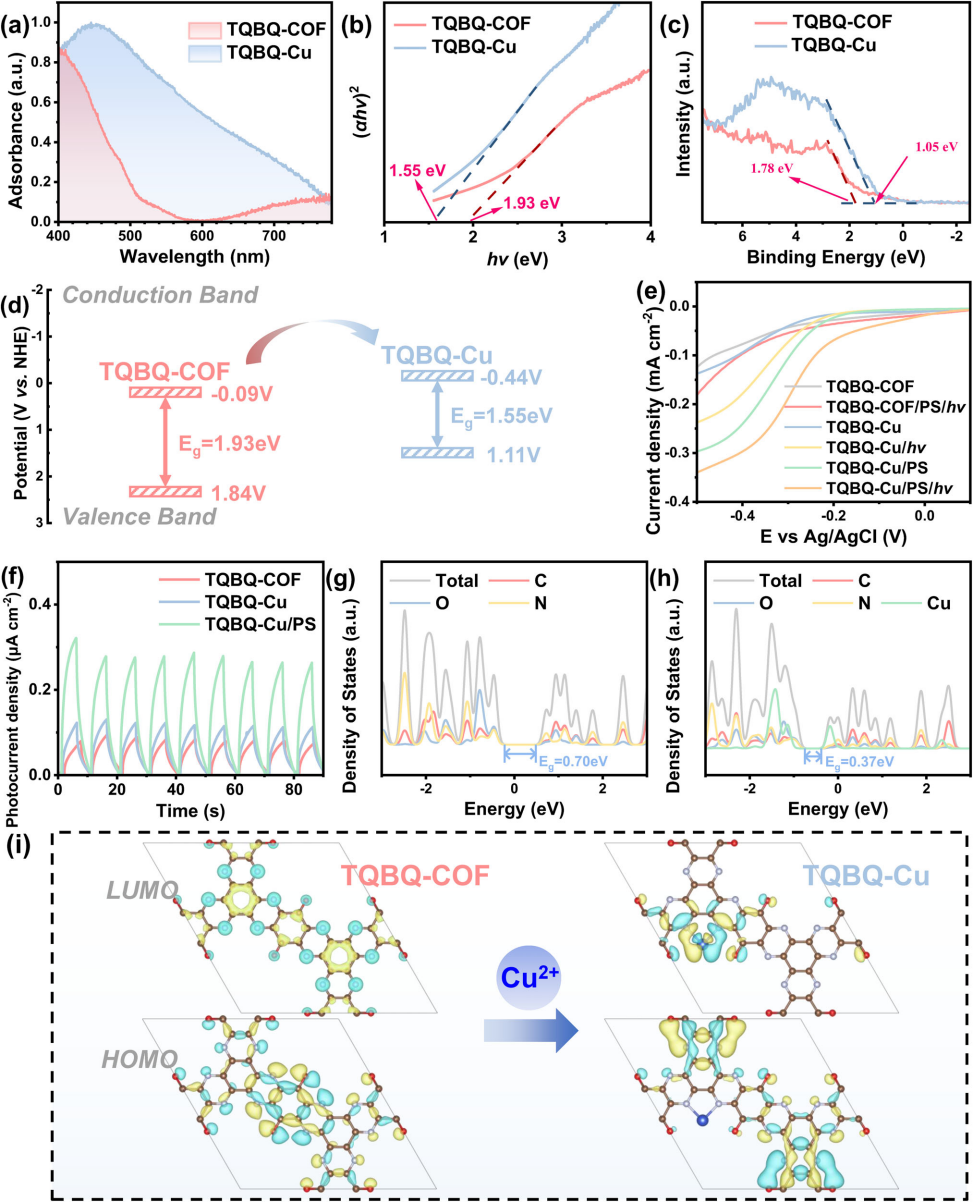
a) UV–vis–NIR DRS spectra, b) Tauc plots, c) XPS VB spectra, and d) illustration of electronic band structure of TQBQ‐COF and TQBQ‐Cu. e) LSV curves of TQBQ‐COF and TQBQ‐Cu and under different conditions. f) Photocurrent response comparisons. g,h) Density of states and i) HOMO/ LUMO diagram of TQBQ‐COF and TQBQ‐Cu calculated by DFT calculation. ([BPA] = 20 ppm, [PS] = 1.5 mm unless specified, [catalyst] = 0.1 g L^−1^).

As shown in Figure 1c,d, TQBQ‐Cu exhibited both excellent PS activation and VL utilization capability. To explore the source of the superior photo‐Fenton performance of TQBQ‐Cu, its optical properties were compared with the original TQBQ‐COF. The UV–vis diffuse reflectance spectra (DRS, **Figure** **3**a) clearly illustrated that the TQBQ‐COF established a single absorption peak at ≈390 nm, attributed to π‐π^*^ electron transitions of the graphene‐like sp^2^ hybridization structure.^[^ ^41^ ^]^ Upon Cu coordination, TQBQ‐Cu retained this π‐π^*^ transition peak with a redshift and demonstrated markedly enhanced light absorption across the entire 200–800 nm. According to the corresponding Tauc plots, the bandgap energies (E_g_) of TQBQ‐COF and TQBQ‐Cu were calculated to be 1.93 and 1.55 eV, respectively (Figure 3b). Moreover, the valence band (VB) positions were measured by XPS VB spectra (Figure 3c; Equation S1, Supporting Information). The overall results are illustrated in Figure 3d. Additionally, both the COFs exhibited negligible photoluminescence signal, suggesting a low recombination rate of photogenerated carriers. The results revealed that Cu coordination significantly modulated the electronic structure, leading to a narrower bandgap and broader light absorption, thereby enhancing photocatalytic performance.

To further explore the photoelectrochemical differences between TQBQ‐COF and TQBQ‐Cu, a series of electrochemical measurements was conducted. Electronic impedance spectroscopy (EIS, Figure S23, Supporting Information) revealed that TQBQ‐Cu had a lower charge transfer resistance than TQBQ‐COF, indicating superior conductivity and charge mobility. The linear sweep voltammetry (LSV) curves (Figure 3e) and light‐chop photocurrent response tests (Figure 3f) were employed to assess the electron transfer process. The TQBQ‐COF exhibited the flattest slope of the LSV curve, with negligible changes under visible light irradiation and PS addition, indicating weak electrochemical performance and merely little response to visible light and PS. In contrast, TQBQ‐Cu showed a significant increase in current density after PS addition and visible light irradiation, indicating that Cu could enhance charge separation and migration, and improve PS activation activity during photo‐Fenton degradation.

To gain deeper insight into the enhanced catalytic activity of TQBQ‐Cu at the molecular level, DFT calculations were subsequently carried out. As shown in the calculated band structure and density of states (DOS), the bandgap of TQBQ‐COF significantly narrowed from 0.70 to 0.37 eV after Cu doping, which aligned well with the experimental values derived from Tauc plots (Figure 3g,h). The spatial distributions of highest occupied molecular orbitals (HOMOs) and lowest unoccupied molecular orbitals (LUMOs) were also analyzed (Figure 3i). TQBQ‐Cu exhibited a significant separation distribution between HOMO and LUMO regions compared to TQBQ‐COF, which could effectively promote excitons dissociation and charge transfer.^[^ ^42^ ^]^

Overall, the experimental and computational results confirm that Cu incorporation plays a critical role in modulating the HOMO/LUMO structure and therefore boosting the photo‐Fenton reaction of the system by significantly enhancing the visible light absorption and photocarrier separation capabilities of the catalyst.

### 2.5 Degradation Mechanism Investigation

**Figure 4:**
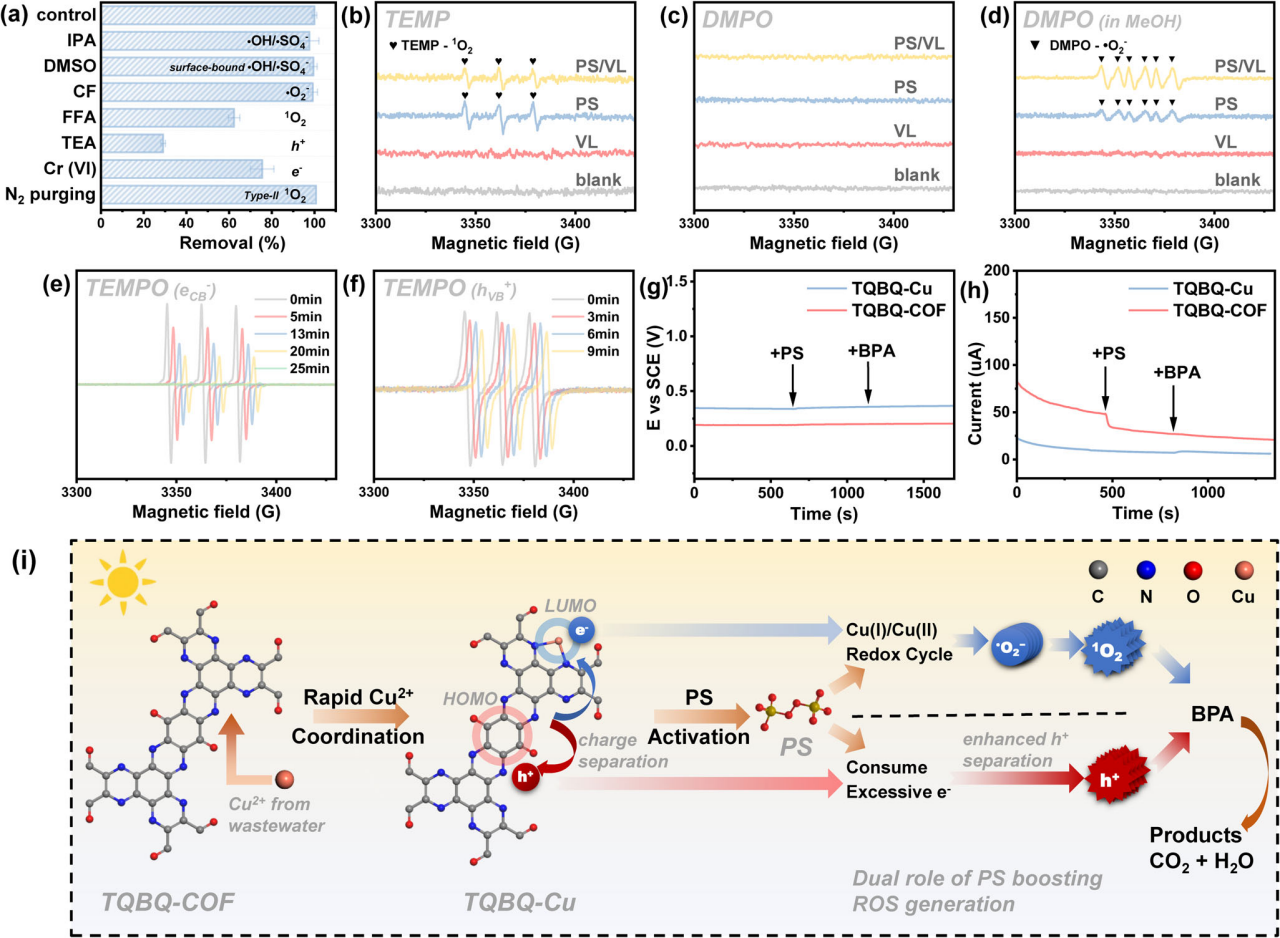
a) BPA degradation rate by TQBQ‐Cu/VL/PS systems under different quenchers conditions within 60 min. EPR spectra of TQBQ‐Cu under different condition with addition of b) TEMP in water, c) DMPO in water, d) DMPO in methanol, e) TEMPO in water and f) TEMPO in acetonitrile (*The x‐axis (Magnetic field) in Figure 4e,f is referenced to the spectra at 0 min, with later time points shown with an incremental offset of 0.3 G per spectrum for clarity*.). g) Open‐circuit potential curves on TQBQ‐Cu‐loaded glassy carbon electrodes after addition of 1.5 mm PS and 20 ppm BPA. h) I‐t curves of the catalysts in response to PS and PhOH addition. i) Proposed mechanism of Cu incorporation and PS boosting photo‐Fenton process. ([BPA] = 20 ppm, [PS] = 1.5 mm, [catalyst] = 0.1 g L^−1^, [IPA] = 5 mm, [DMSO] = 10 mm, [CF] = 120 mm, [FFA] = 5 mm, [TEA] = 5 mm, [Cr (VI)] = 0.5 mm if used).

The reactive oxygen species (ROS) involved in the system were initially investigated using quenching experiments (**Figure** **4**a; Figures S24 and S25, Supporting Information). Isopropanol (IPA), dimethyl sulfoxide (DMSO), and chloroform (CF) were employed as scavengers for sulfate radical and hydroxyl radical (SO_4_^•−^/•OH), surface‐bounded SO_4_^•−^ and •OH, and superoxide radicals (•O_2_^−^), respectively (Figure 4a; Table S2, Supporting Information).^[^ ^43^, ^44^, ^45^, ^46^ ^]^ As a result, IPA, DMSO, and CF caused negligible inhibition of the TQBQ‐Cu/PS/VL system, indicating that •OH, SO_4_^•−^, and •O_2_^−^ were not the dominant active species. For non‐radical oxidation pathways, the partial inhibition by furfuryl alcohol (FFA), a ^1^O_2_ quencher, confirmed the involvement of ^1^O_2_ in the BPA degradation process under the TQBQ‐Cu/PS/VL system.^[^ ^47^ ^]^ Moreover, triethanolamine (TEA) and potassium dichromate (K_2_Cr_2_O_7_) were introduced as quenchers for photo‐generated *h* ^+^ and *e* ^−^, respectively.^[^ ^48^ ^]^ The inhibition observed upon their addition confirmed the contribution of photogenerated *h* ^+^ and *e* ^−^ in the photocatalytic degradation process.

To further confirm the presence of activate species and provide additional insight into the reaction mechanism, electron paramagnetic resonance (EPR) analysis was performed (Figure 4b–d). The triplet TEMP‐^1^O_2_ peaks with an intensity ratio of 1:1:1 was observed, supporting the involvement of ^1^O_2_ in the degradation process. While no signal was detected corresponding to the DMPO‐•OH or DMPO‐SO_4_^•−^, which is consistent with the results from the quenching experiments. However, upon introduction of PS or PS/VL into the TQBQ‐Cu methanol solution, the sextuple peaks of DMPO‐•O_2_^−^ were detected, indicating the generation of •O_2_^−^ under these conditions. The negligible inhibition observed with CF suggested that •O_2_^−^ may rapidly convert to ^1^O_2_ on the catalyst surface, thereby acting as an intermediate species in the overall reaction pathway.^[^ ^49^ ^]^ The participation of photogenerated *h* ^+^ and *e^−^* in TQBQ‐Cu /PS/VL system was also identified by monitoring the TEMPO signal in acetonitrile (detecting *h* ^+^) and water (detecting *e* ^−^) solution (Figure 4e,f).^[^ ^16^ ^]^ Additionally, the potential involvement of electron‐transfer process (ETP) in the PS activation was examined through open‐circuit potential analysis and amperometric i‐t curves (Figure 4g,h).^[^ ^50^, ^51^, ^52^ ^]^ No significant signal change was observed after the addition of PS and BPA, indicating that ETP was not a significant contributor to the degradation process.

Subsequently, the origin of ^1^O_2_ and activation of PS in this system were investigated. In the absence of PS, the photogeneration of ^1^O_2_ was classified by Gollnick into Type I and Type II reactions.^[^ ^53^, ^54^, ^55^ ^]^ Type I photosensitization reaction features electron‐transfer process, in which after electron‐hole separation, highly reactive radicals are produced in advance through electron transfer from the *h* ^+^/*e^−^*, then ^1^O_2_ is generated through a series of reactions of radicals (Equations S2–S10, Supporting Information).^[^ ^56^, ^57^, ^58^, ^59^ ^]^ Whereas Type II reaction only undergoes energy‐transfer process from the photochemical excited photosensitizer to dissolved oxygen in the solution to produce ^1^O_2_ (Equations S11–S13, Supporting Information).^[^ ^60^, ^61^ ^]^ Amongst them, Type I photogeneration was excluded by the absence of •OH radical. To verify the contribution of the Type II ^1^O_2_ photogeneration, nitrogen (N_2_) was continuously purged into the system to remove the dissolved oxygen. The degradation performance was not affected by N_2_ purging, excluding the Type II reaction in this system (Figures 4a and S24b, Supporting Information). EPR analysis further proved that the TQBQ‐Cu could not generate ^1^O_2_ by light irradiation without PS addition (Figure 4b). This also explained why TQBQ‐Cu was unable to degrade BPA under visible light irradiation without PS, proving strong evidence for the exclusion of these two photogeneration pathways. Therefore, the ^1^O_2_ was most likely to be generated directly through the activation of PS, but not transformed from •O_2_^−^ through photocatalysis reactions. The possible PS activation pathways were presented in Equations S14–S17 (Supporting Information).^[^ ^62^, ^63^, ^64^ ^]^ In addition, the interaction between PS and TQBQ‐Cu was predicted using DFT calculations (Figure S26, Supporting Information). The results showed that PS was prone to being adsorbed at the Cu sites, which is the LUMO position in the structure where it is easiest to donate electrons to PS (Equations S15–S17, Supporting Information). Therefore, based on the comprehensive characterization results and theoretical calculations, an activation pathway was proposed for the effective photo‐Fenton process under visible light irradiation and PS addition (Equations (1), (2), (3), (4), (5), (6), (7), (8) and Figure 4i).

(1) $$\mathrm{TQBQ}-\mathrm{Cu}+\mathrm{VL}\to {e_{CB}}^{-}+{h_{VB}}^{+}$$

(2) $$\mathrm{Cu}\left(I\right)+S_{2}O_{8}^{2-}+H_{2}O\;\to \;\mathrm{Cu}\left(I\right)-{\mathrm{OOSO}}_{3}^{2-}+{\;\mathrm{SO}}_{4}^{2-}+2H^{+}$$

(3)

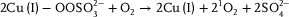

(4) $$\mathrm{Cu}\left(I\right)+S_{2}O_{8}^{2-}+2H_{2}O\to \mathrm{Cu}\left(\mathrm{II}\right)+•{O_{2}}^{-}+2{\mathrm{SO}}_{4}^{2-}+4H^{+}$$

(5) $$\mathrm{Cu}\left(\mathrm{II}\right)+{e_{CB}}^{-}\to \mathrm{Cu}\left(I\right)$$

(6)

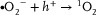



(7)

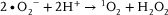



(8)

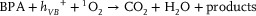




In conclusion, *h* ^+^ and ^1^O_2_ were the main active species in TQBQ‐Cu/PS/VL degradation system. ^1^O_2_ was generated by activation of PS (Equations (2), (3), (4), (5)), while *h* ^+^ was generated by visible light irradiation (Equation 1). Both the Cu and PS played crucial roles in this photo‐Fenton process. Cu significantly alters the electronic structure of TQBQ‐COF, leading to a substantial improvement in the catalyst's photoreaction performance. Meanwhile, Cu serves as a sustainable electron‐donating site, causing PS activation to generate ^1^O_2_. On the other hand, PS significantly enhanced the photogenerated *h* ^+^ production by consuming the excessive e^−^ and promoting charge separation. As a result, the in situ coordination of Cu realized a substantial progression from negligible to excellent degradation performance of the system.

### 2.6 Degradation Pathway and Practical Application Investigation

Sometimes highly toxic intermediates or by‐product may be produced during the decontamination process.^[^ ^65^ ^]^ To evaluate the degradation pathway and overall toxicity during the process, the degradation intermediates were detected using LC‐Q‐TOF mass spectrometer. In total, 13 intermediates were identified according to both the precise m/z values and previous literature (Figure S27 and Table S3, Supporting Information).^[^ ^17^, ^66^ ^]^ To rationally analyze the degradation pathways, the DFT calculation on the BPA molecule was conducted to predict the reactive sites for degradation. As indicated by the HOMO isotherm of BPA (Figure S28, Supporting Information), the benzene ring and hydroxyl may be the most electron rich sites. The active sites of BPA were predicted by the f^−^ value of Fukui function. (Table S4, Supporting Information) A higher f^−^ value indicates the higher possible reactivity of the atom with electrophilic reactive species (^1^O_2_ and *h* ^+^).^[^ ^67^ ^]^ Overall, the phenolic ring of BPA is more reactive in this system, possibly resulting in easier ring‐opening reactions by the attack of *h* ^+^.^[^ ^68^ ^]^ The possible degradation pathways were proposed in Figure S28 (Supporting Information). The effective ring‐opening reactions during the degradation were in accordance with the electrophilic feature of the ROS. To further and more comprehensively evaluate the availability and environmental‐friendliness of this degradation system, ECOSAR was applied to assess the ecotoxicity of the degradation by‐products. According to the aquatic toxicity classification by the United Nations Globally Harmonized System, the substances with short‐term or chronic hazard lg(LC_50_/EC_50_ or ChV) < 0 toward fish, crustacea, or algae are considered very toxic, while that with lg(LC_50_ or EC_50_) > 2 are not harmful.^[^ ^69^ ^]^ As depicted in the individual heatmap under every substance in Figures S28 and S29 (Supporting Information), all the intermediates exhibited lower toxicity than the original BPA. Moreover, as the degradation progresses, the overall toxicity was reduced rapidly by the ring‐opening reactions and step by step, until the organics transform into non‐toxic aliphatic molecules or undergo complete mineralization.

**Figure 5:**
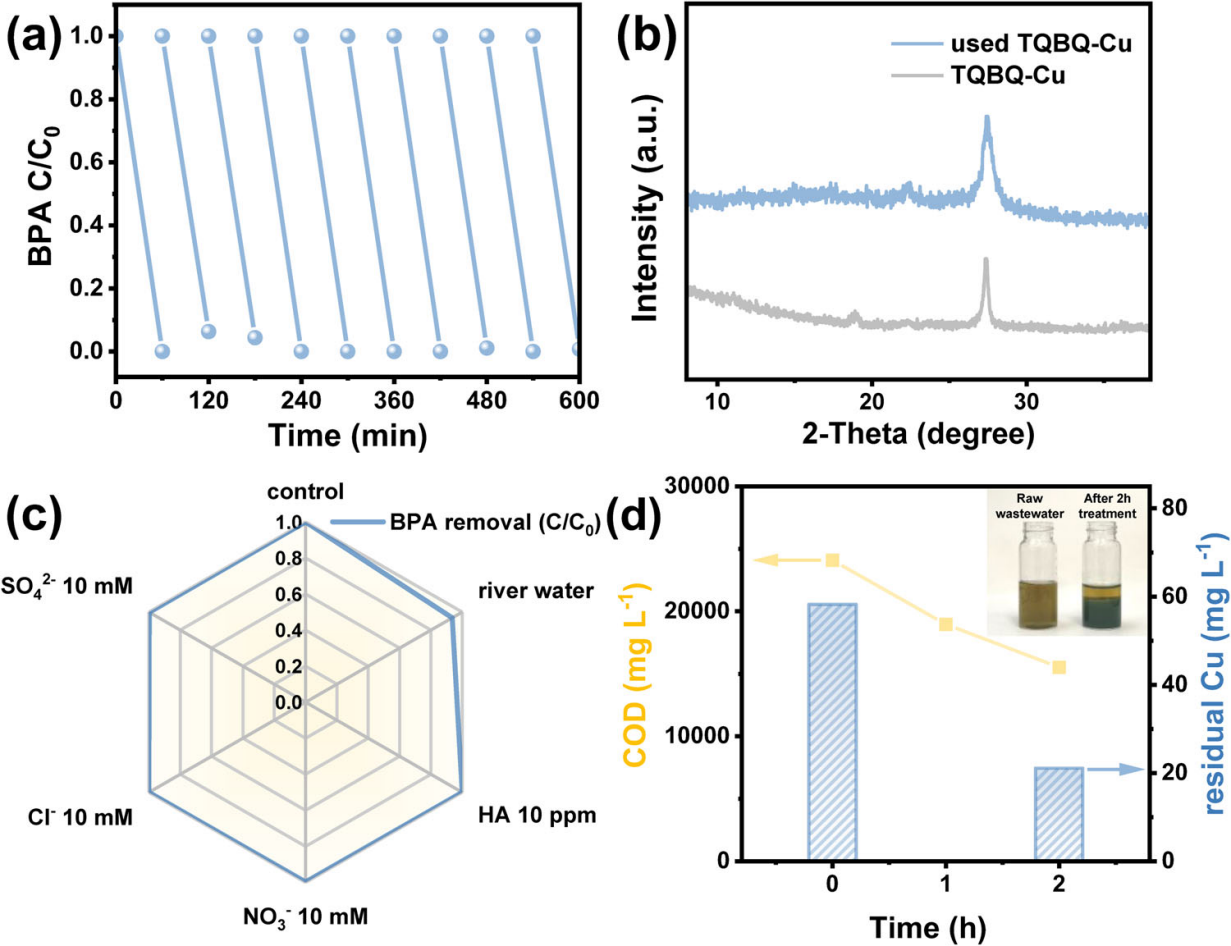
a) Cycle test of TQBQ‐Cu/VL/PS for BPA removal. b) XRD pattern of fresh and used catalysts. c) BPA degradation rate by TQBQ/VL/PS systems with the existence of different environmental substances. ([BPA] = 20 ppm, [PS] = 1.5 mm, [catalyst] = 0.1 g L^−1^) d) COD removal by TQBQ‐COF/VL/PS system and residual Cu concentration after 0.1 m NaOH precipitation. (inset: image of the precipitate by 0.1 m NaOH) ([TQBQ‐COF] = 0.3 g L^−1^, [PS] = 15 mm).

The long‐term stability is another important factor for the catalyst. The catalytic stability of TQBQ‐Cu was examined by the recycling experiments. As presented in **Figure** **5**a, the catalysts were reused for 10 cycles. After 10 rounds, the degradation rate could still maintain > 90%, demonstrating the outstanding stability and practicability of the TQBQ‐Cu. Moreover, the crystallinity of the used TQBQ‐Cu was also monitored by XRD patterns (Figure 5b). The negligibly changed peak at 27.4° indicated the robustness of the carbon skeleton. Moreover, selective degradation enhances the utilization efficiency of the oxidant and ROS while avoiding undesired degradation by‐products, especially in the presence of common environmental substances such as ions and natural organic matters. The better selectivity toward electron‐rich pollutants is a major advantage of non‐radical pathway‐dominated degradation systems.^[^ ^70^ ^]^ To quantify this benefit, the anti‐interference ability of this system was evaluated with the existence of CO_3_^2−^, NO_3_^2−^, Cl^−^, SO_4_^2−^, humic acid (HA), or in tap water and river water.^[^ ^71^ ^]^ As shown in Figure 5c and Figure S30 (Supporting Information), the degradation was negligibly affected by the interfering substances, indicating its good selectivity and anti‐interference performances. This, in turn, also corroborates the non‐radical mechanism of this system, ensuring its efficacy in practical wastewater treatment applications.

To further verify the feasibility of this in situ Cu utilization system, real wastewater experiments were conducted using an effluent sample from a copper electroplating factory in Chengdu, Sichuan. The detailed information of the wastewater was provided in Table S5 (Supporting Information). As presented in Table S6 (Supporting Information), upon addition of TQBQ‐COF, 9.2 mm Cu was adsorbed onto the material, ensuring its subsequent availability for catalytic utilization. After 2 h of photo‐Fenton process, the total COD decreased significantly by 8560 mg L^−1^ (35%) (Figure 5d), demonstrating the system's efficient Cu utilization and high organic degradation capability. Moreover, in real wastewater, Cu usually exists as metal‐organic complexes with various organic ligands (e.g., EDTA, nitrilotriacetic acid). The strong complexes resist chemical precipitation until the organics are destroyed and free Cu ions are released.^[^ ^9^, ^72^, ^73^, ^74^ ^]^ For the original copper electroplating effluent sample, no precipitate was observed after 0.1 m NaOH addition (Figure 5d inset). After treating 2 h with the TQBQ‐COF/PS/VL system, the Cu removal efficiency reached 63.0%, indicating a significantly enhanced decomplexation of metal‐organic species and improved recoverability of Cu (Table S6, Supporting Information).

## 3 Conclusion

In this work, the co‐existing Cu^2+^ in wastewater was successfully in situ utilized for boosting Fenton‐like performance, by a 2D π‐conjugated TQBQ‐COF with abundant pre‐designed 2N metal anchoring sites. The single dispersed Cu sites could greatly modulate the electronic structure and enhance the visible‐light‐absorption activity of the COF, promoting the charge carrier separation efficiency and PS activation, therefore, remarkably improve the catalytic Fenton‐like performance. Quenching experiments and EPR experiments identified ^1^O_2_ and *h* ^+^ as the main active species in this system. The degradation pathway was systematically analyzed, excluding the formation of toxic intermediates during pollutant degradation. Moreover, the catalyst is highly effective in promoting the organic pollutant degradation and Cu^2+^ removal simultaneously, even in actual electroplating wastewater containing ultrahigh concentration of Cu (> 4000 ppm) and COD (> 20 000 ppm), demonstrating the strong applicability of this strategy for real wastewater decontamination. This mild post‐modification strategy provides a generalizable design strategy of “treating waste with waste” and inspirations for catalyst design, water treatment, and environmental remediation.

## Conflict of Interest

The authors declare no conflict of interest.

## Supporting information



Supporting Information

## Data Availability

The data that support the findings of this study are available from the corresponding author upon reasonable request.
