# Supplementary material for: In Situ Utilization of Co‐Existing Metal Ions to Fabricate Single‐Atom Catalyst for Boosting Fenton‐Like Activity
Source: Adv Sci (Weinh). 2025 Dec 8;13(4):e11761. doi: 10.1002/advs.202511761 (PMC12822421; doi:10.1002/advs.202511761)
Supplement: Supplementary file 1 — Supporting Information [file ADVS-13-e11761-s001.pdf]

<u>Supporting information for</u>

In-situ utilization of co-existing metal ions to fabricate single-atom catalyst for boosting Fenton-like activity

Jiaxing Yu [a], Shaohan Wang [a], Huajie Zhong [b], Zeyu Gong [b], Yuan Tao [b,*], Junhui Wang [b,*], Zhengping Hao [c], Gangfeng Ouyang [a, b, d, e]

[a] MOE Key Laboratory of Bioinorganic and Synthetic Chemistry/KLGHEI of Environment and Energy Chemistry, School of Chemistry, Sun Yat-Sen University, No. 135, Xingang Xi Road, Guangzhou, Guangdong, 510275, China

[b] School of Chemical Engineering and Technology, Sun Yat-Sen University, Zhuhai 519087, China

[c] National Engineering Laboratory for VOCs Pollution Control Material & Technology, University of Chinese Academy of Sciences, Beijing 101408, China

[d] Chemistry College, Center of Advanced Analysis and Gene Sequencing, Zhengzhou University, Zhengzhou 450001, China

[e] Provincial Key Laboratory of Emergency Test for Dangerous Chemicals, Guangdong Provincial Engineering Research Center for Ambient Mass Spectrometry, Institute of Analysis, Guangdong Academy of Sciences (China National Analytical Center Guangzhou), 100 Xianlie Middle Road, Guangzhou 510070, China

* Corresponding author:

Tel: +86 020 84110845/0953; fax: +86 020 84110845/0953.

E-mail address: wangjh36@mail.sysu.edu.cn (J. Wang); taoy58@mail.sysu.edu.cn (Y. Tao)

Supplementary Methods

Materials.

The following reagents were used as received: Hexaketocyclohexane (HKH, octahydrate, 99%) were purchased from Energy Chemical. Tetramino-benzoquinone (TABQ) were acquired from Bidepharm, N-Methylpyrrolidone (NMP), copper (II) sulfate (anhydrate, 99.0%), BPA (CP grade), PS (99.99%), isopropanol (IPA), dimethyl sulfoxide (DMSO), chloroform (CF), furfuryl alcohol (FFA), triethanolamine (TEA), humic acid (HA) and 2,2,6,6-Tetramethyl-4-piperidinol (TEMP, ≥98%) were purchased from Aladdin, potassium dichromate (Cr (VI), AR) was purchased from Tianjin Damao, 5,5-dimethyl-1-pyrrolidine N-oxide (DMPO, 98%) were obtained from Dojindo. Milli-Q ultrapure deionized water (≥18 MΩ·cm) was used throughout the whole work.

Synthesis of TQBQ-COF and TQBQ-Cu.

The synthesis of TQBQ-COF was conducted following a modified method from Wu *et al.*[1] 347.6 mg HKH (0.4 mmol) and 151.2 mg TABQ (0.6 mmol) was precisely weighed into a 30mL pyrex tube under an ice bath. The atmosphere was replaced with pure nitrogen, then 15 mL deoxygenated N-methyl-2-pyrrolidone containing 0.5 mL sulfuric acid was slowly added. The mixture was gently stirred for 3.5 h under room temperature, then heated to 75 °C for 12 h. Finally, the product was washed out with methanol and separated by filtration and then washed by water and ethanol using Soxhlet extractor. The obtained COF was dried at 80 °C under vacuum for 12 h.

Ex-situ fabricated TQBQ-Cu (for characterization) was synthesized by simply adding 0.1 g/L COF and 0.5 mM $CuSO_4$ into deionized water. After 12 h of stirring under room temperature, the obtained TQBQ-Cu was washed by methanol and water, then dried at 80 °C under vacuum for 12 h.

Experimental procedure.

All photocatalytic reactions were performed using a multi-channel photochemical reactor (Perfectlight PCX-50C) in separate 50 mL quartz bottles (Shimazu) maintained at 25 °C. The system was equipped with a 10 W white LED light source ($800 \geq \lambda \geq 400$ nm) serving as simulated visible light, which was positioned to irradiate the vials from the bottom. The light intensity was calibrated to ~100 mW/cm². The stirring was kept 300 rpm during the reactions. In a typical experiment, the reaction was carried out in 20 mL solution with certain concentration of the catalyst and BPA (and Cu^{2+}). After magnetically stirring in the dark for at least 20 min to establish the BPA (and Cu^{2+}) adsorption-desorption equilibrium, the reaction was initiated by adding PS solution and/or VL irradiation. Samples were extracted using a 1 mL syringe and filtered subsequently (0.22 μm PTFE filter) at certain time points during the reaction. To quench the excessive oxidant, 20uL Na_2SO_3 was added into each 2-mL sample vial.

Characterization.

The electrochemical experiments were conducted using CH instrument CHI760E workstation. The XRD spectra were measured by a Rigaku SmartLab system. The UV-vis-NIR DRS spectra were acquired on a Shimazu UV-3600 spectrometer. The EPR spectra were obtained on a CW/Pulse EPR system (A300, Bruker Co., Germany) with a microwave frequency of 9.64 GHz, a microwave power of 0.94 mW, a modulation frequency of 100 kHz, and a modulation amplitude of 2.0 G. FT-IR spectra were acquired on a PerkinElmer Frontier. Raman spectra were recorded on a Renishaw InVia spectrometer with a model 100 Ramascope optical fiber instrument. X-ray photoelectron spectroscopy (XPS) analysis was obtained on an ESCALAB 250 spectrometer (Thermo Fisher Scientific Corporation, USA) with a 150W Al Kα radiation as the excitation source. The spectra were calibrated so that the C 1s sp3-C peak is at 284.8 eV. The component fitting for C 1s, O 1s, N 1s and Cu 2p spectra were proceeded with Gaussian-Lorentzian (20:80) components using Shirley background by XPSPeak41 software. The Cu concentration in aqueous samples were measured on a Jena contrAA 800 AAS spectrometer. The chemical oxygen demand (COD)

concentrations were measured by a Lianhua 5B-3C (V8) COD spectrometer. The TOC concentration was measured by a Shimazu TOC-L analyzer.

Analysis method

The concentrations of the target organic compound were analyzed using a high-performance liquid chromatography (HPLC, Shimadzu LC-20AD). Separation was performed on a Poroshell 120 EC-C18 column (4.6×100 mm, 2.7 μm, Agilent Technology, USA) using a mobile phase consisting of a binary mixture of water and methanol at a flow rate of 0.6 mL/min. The BPA concentration was measured at UV length of 226 nm via standard curve, with methanol/water solvent ratio of 70:30.

Calculation of pseudo-first order rate constant k_{obs}

The first order kinetic reaction follows a first order rate equation as below.

$$Rate = -\frac{d\,c}{d\,t} = k_{obs}c$$

$$ln\frac{c}{c_0} = k_{obs}t$$

c and c_0 are the concentration of substituent at t and initial time. The natural logarithm of the relative concentration graphically plotted against time results in a straight line with a slope of k_{obs}.

Supplemental equations (Eqs.)

The valence band potential ($E_{VB,\ NHE}$) calculated from the potential acquired by XPS VB spectra ($E_{VB,\ XPS}$):

$E_{VB,\ NHE} = \varphi + E_{VB,\ XPS} - 4.44$ ($\varphi = 4.5$ eV) (S1)

Type I photosensitization reactions:

$e^{-} + O_2 \rightarrow \bullet O_2^{-}$ (S2)

$2\bullet O_2^{-} + 2H^{+} \rightarrow O_2 + H_2O_2$ (S3)

$\bullet O_2^{-} + H_2O_2 \rightarrow O_2 + H_2O + \bullet OH$ (S4)

$\bullet OH + H_2O_2 \rightarrow \bullet O_2^{-} + H_2O + H^{+}$ (S5)

$e^{-} + H_2O_2 \rightarrow \bullet OH + OH^{-}$ (S6)

$2\bullet O_2^{-} + 2H^{+} \rightarrow {}^1O_2 + H_2O_2$ (S7)

$\bullet O_2^{-} + h^{+} \rightarrow {}^1O_2$ (S8)

$\bullet O_2^{-} + \bullet OH \rightarrow {}^1O_2 + OH^{-}$ (S9)

$\bullet O_2^{-} + H_2O_2 \rightarrow {}^1O_2 + OH^{-} + \bullet OH$ (S10)

Type II photosensitization reactions:

${}^1Sens + hv \rightarrow {}^1Sens^{*}$ (S11)

${}^1Sens^{*} \xrightarrow{ISC} {}^3Sens^{*}$ (S12)

${}^3Sens^{*} + {}^3O_2 \rightarrow {}^1Sens + {}^1O_2$ (S13)

${}^1O_2$ generation with PS participation:

$Cu(I) + O_2 \rightleftharpoons Cu(II) + \bullet O_2^{-}$ (S14)

$Cu(I) + S_2O_8^{2-} + 2H_2O \rightarrow Cu(II) + \bullet O_2^{-} + 2SO_4^{2-} + 4H^{+}$ (S15)

$Cu(I) + S_2O_8^{2-} + H_2O \rightarrow Cu(I)\text{-}O\text{-}O\text{-}SO_3^{2-} + SO_4^{2-} + 2H^{+}$ (S16)

$2Cu(I)\text{-}O\text{-}O\text{-}SO_3^{2-} + O_2 \rightarrow 2Cu(I) + 2{}^1O_2 + 2SO_4^{2-}$ (S17)

P.S. Type I reactions (S1~S10) may also occur in presence of PS, but for the sake of simplicity, it will not be repeated in this part.

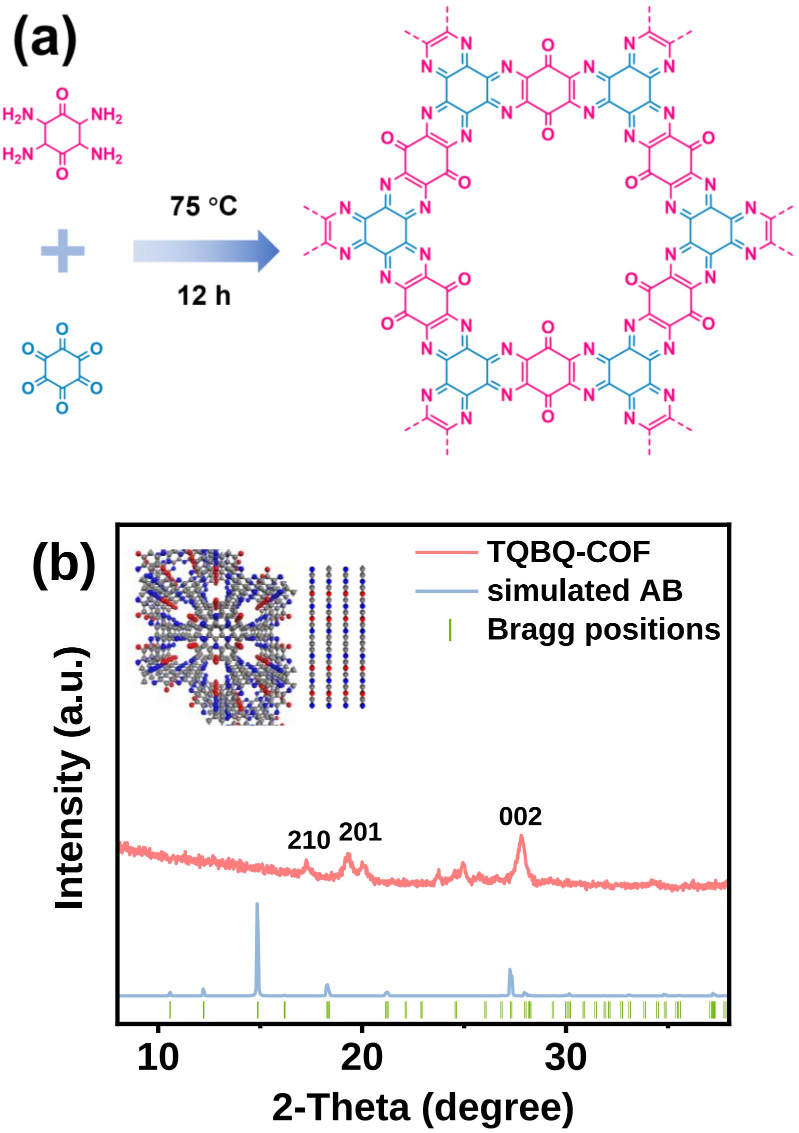

Figure S1. (a) Synthesis route of TQBQ-COF. (b) Powder X-ray diffraction patterns of TQBQ-COF and simulated pattern of TQBQ-COF of AB stacking model.

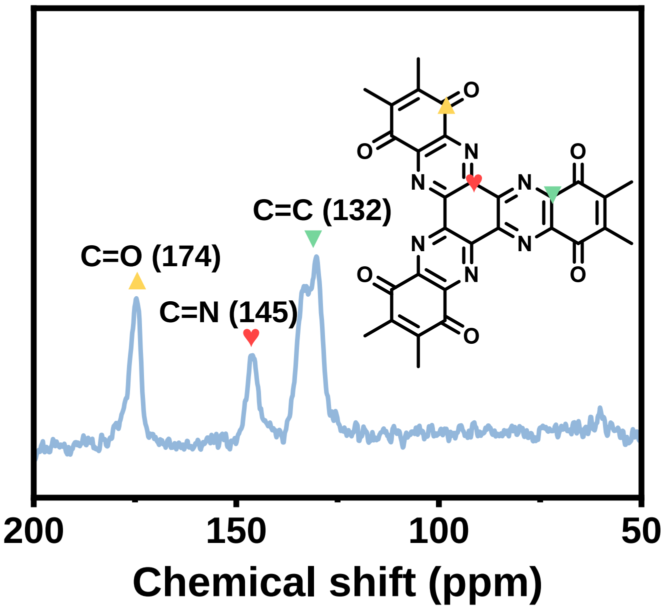

Figure S2. Solid-state ^{13}C nuclear magnetic resonance (NMR) spectroscopy of TQBQ-COF.

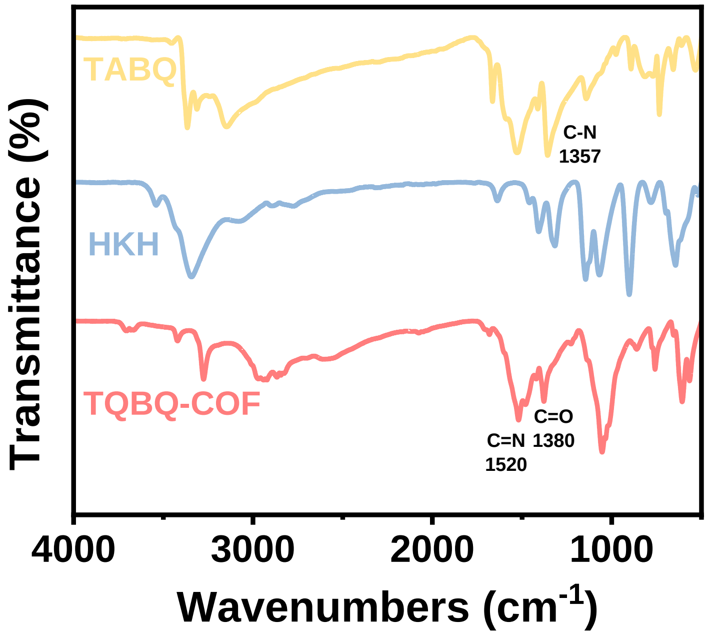

Figure S3. Fourier Transform Infrared (FT-IR) spectra of TABQ, HKH and TQBQ-COF.

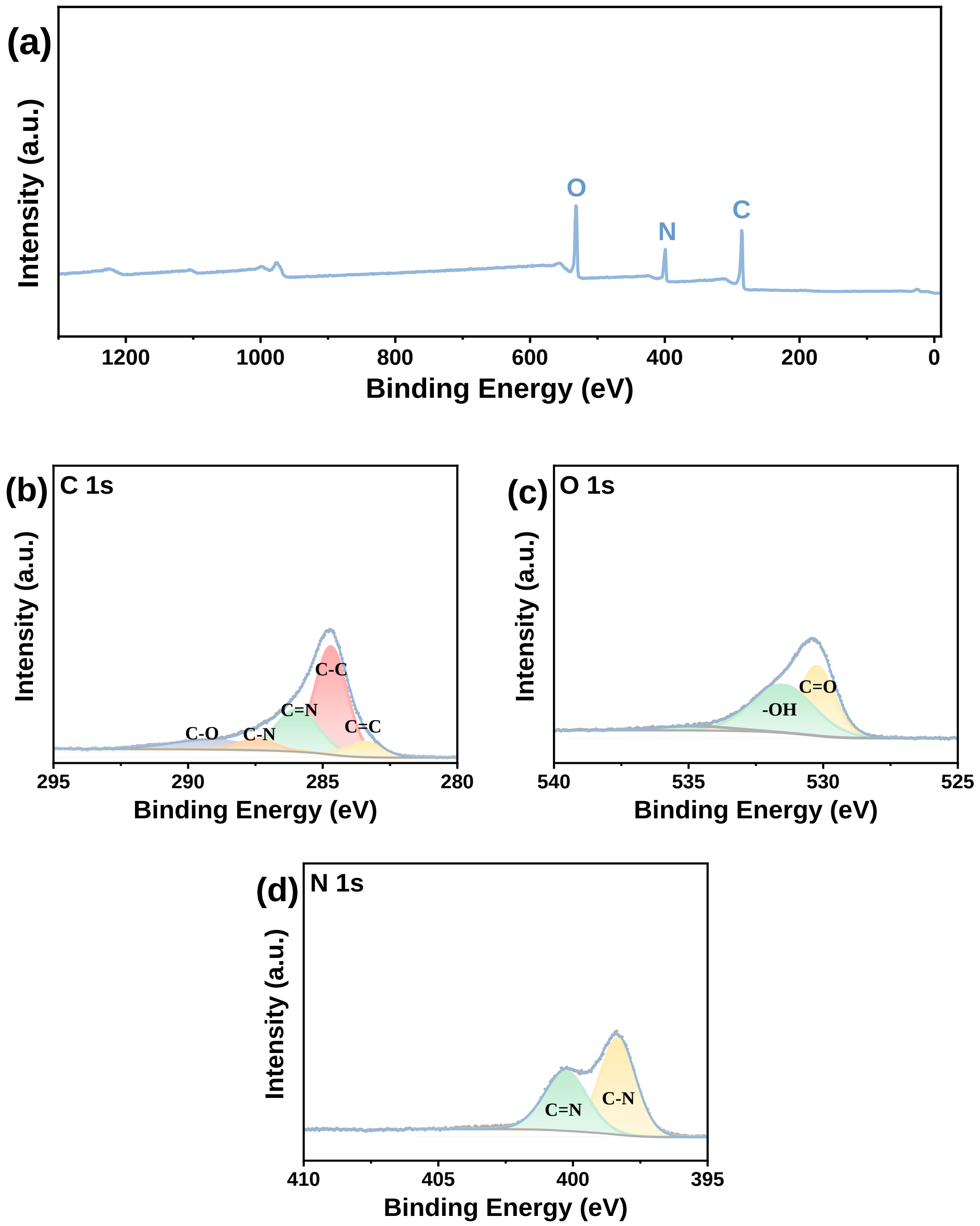

Figure S4. (a) XPS survey spectra of TQBQ-COF. High-resolution XPS (b) C 1s, (c) O 1s and (d) N 1s spectra of TQBQ-COF.

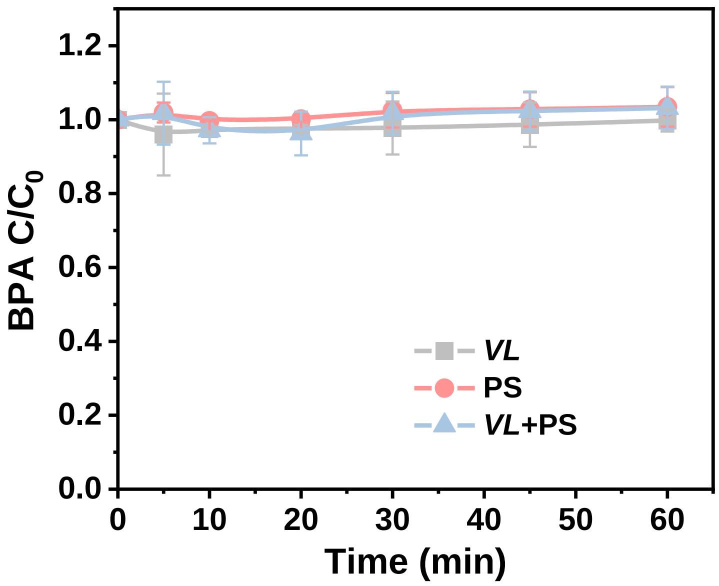

Figure S5. BPA degradation by TQBQ-COF/PS, TQBQ-COF/VL and TQBQ-COF/PS/VL systems. ([BPA] = 20 ppm, [PS] = 1.5 mM if added, [Catalyst] = 0.1 g/L with 0.5 mM Cu^{2+} pre-adsorbed)

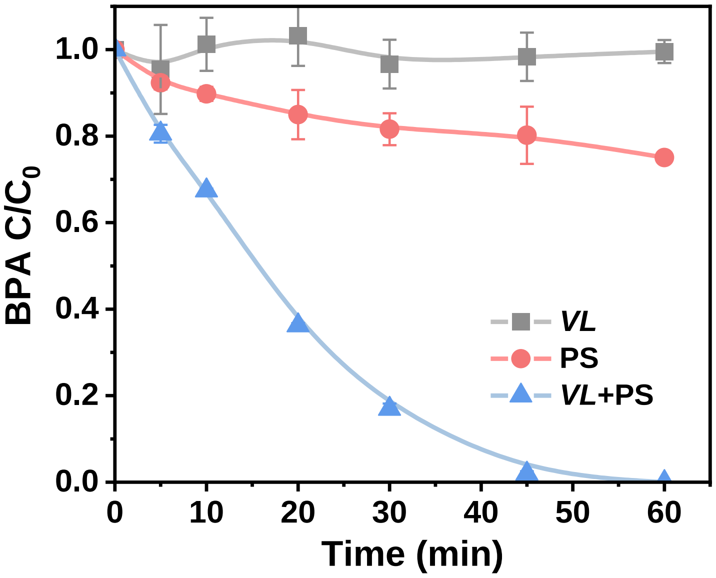

Figure S6. BPA degradation by TQBQ-Cu/PS, TQBQ-Cu/VL and TQBQ-Cu/PS/VL systems. ([BPA] = 20 ppm, [PS] = 1.5 mM if added, [Catalyst] = 0.1 g/L with 0.5 mM Cu^{2+} pre-adsorbed)

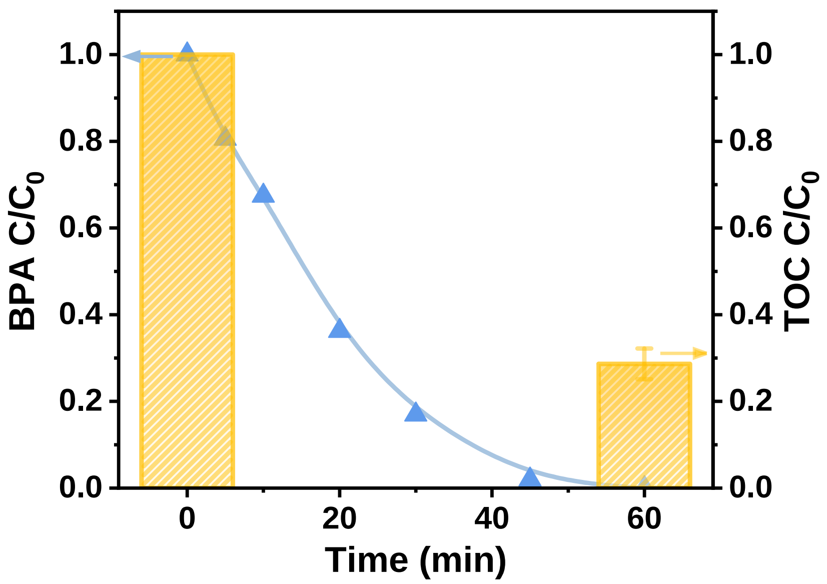

Figure S7. BPA and corresponding total organic carbon (TOC) removal by TQBQ-Cu/PS/VL system. ([BPA] = 20 ppm, [PS] = 1.5 mM, [TQBQ-Cu] = 0.1 g/L with 0.5 mM Cu^{2+} pre-adsorbed)

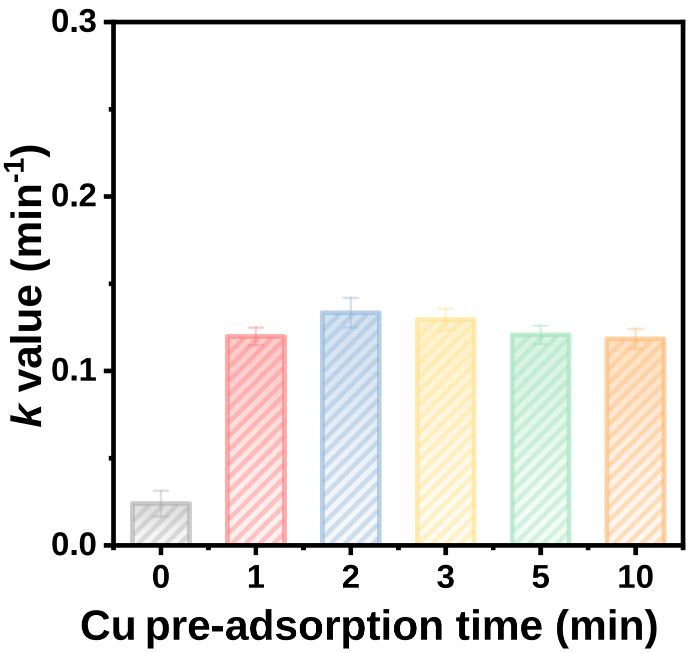

Figure S8. BPA degradation by Cu^{2+}+TQBQ-COF/PS/VL systems after different Cu^{2+} pre-adsorption duration. ([BPA] = 20 ppm, [PS] = 1.5 mM, [Cu^{2+}] = 0.5 mM, [Catalyst] = 0.1 g/L)

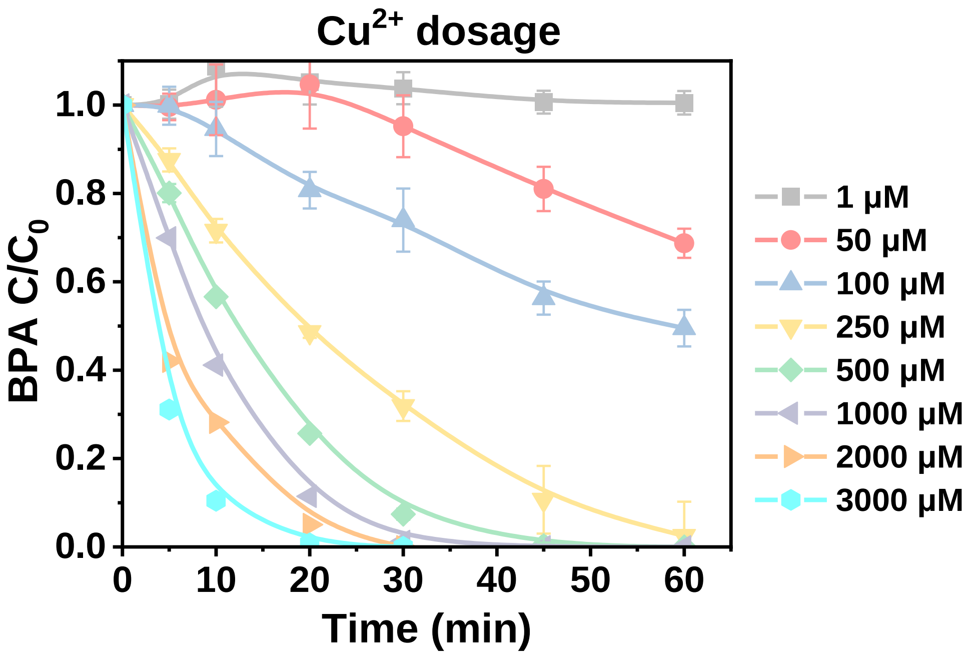

Figure S9. BPA degradation by different Cu^{2+} concentration-doped TQBQ-Cu/PS/*VL* systems. ([BPA] = 20 ppm, [PS] = 1.5 mM if added, [Catalyst] = 0.1 g/L)

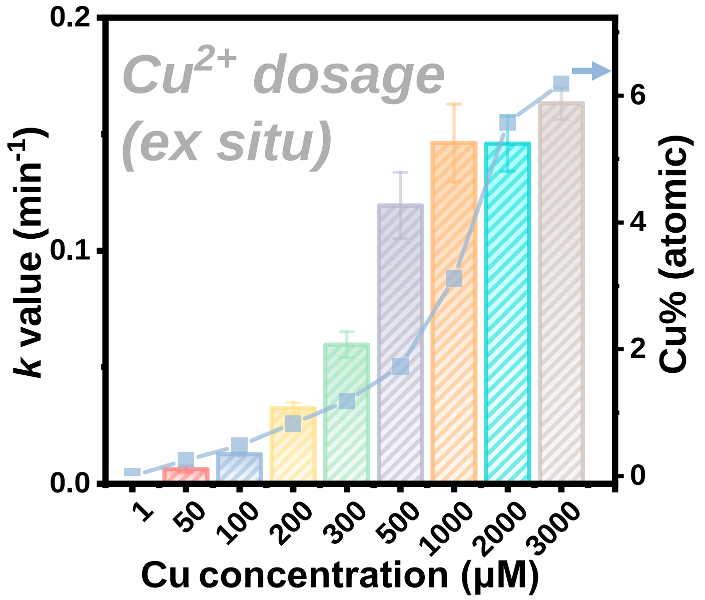

Figure S10. The photo-Fenton efficiency of *ex-situ* prepared TQBQ-Cu with different Cu^{2+} dosage and their atomic Cu^{2+} content characterized by XPS analysis. ([BPA] = 20 ppm, [PS] = 1.5 mM if added, [Catalyst] = 0.1 g/L with 0.5 mM Cu^{2+} pre-adsorbed)

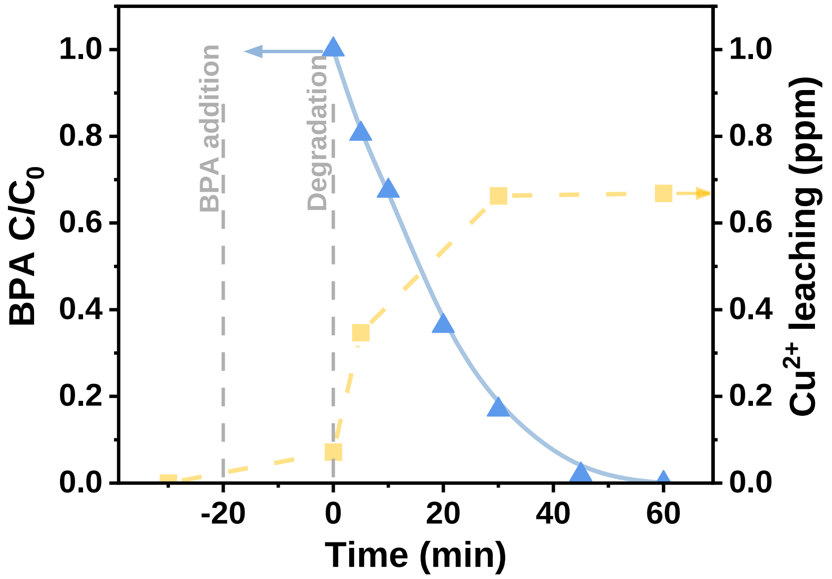

Figure S11. BPA and aqueous Cu^{2+} concentration during the degradation process by *ex-situ* fabricated TQBQ-Cu/PS/VL system. ([BPA] = 20 ppm, [PS] = 1.5 mM, [TQBQ-Cu] = 0.1 g/L with 0.5 mM Cu^{2+} pre-adsorbed)

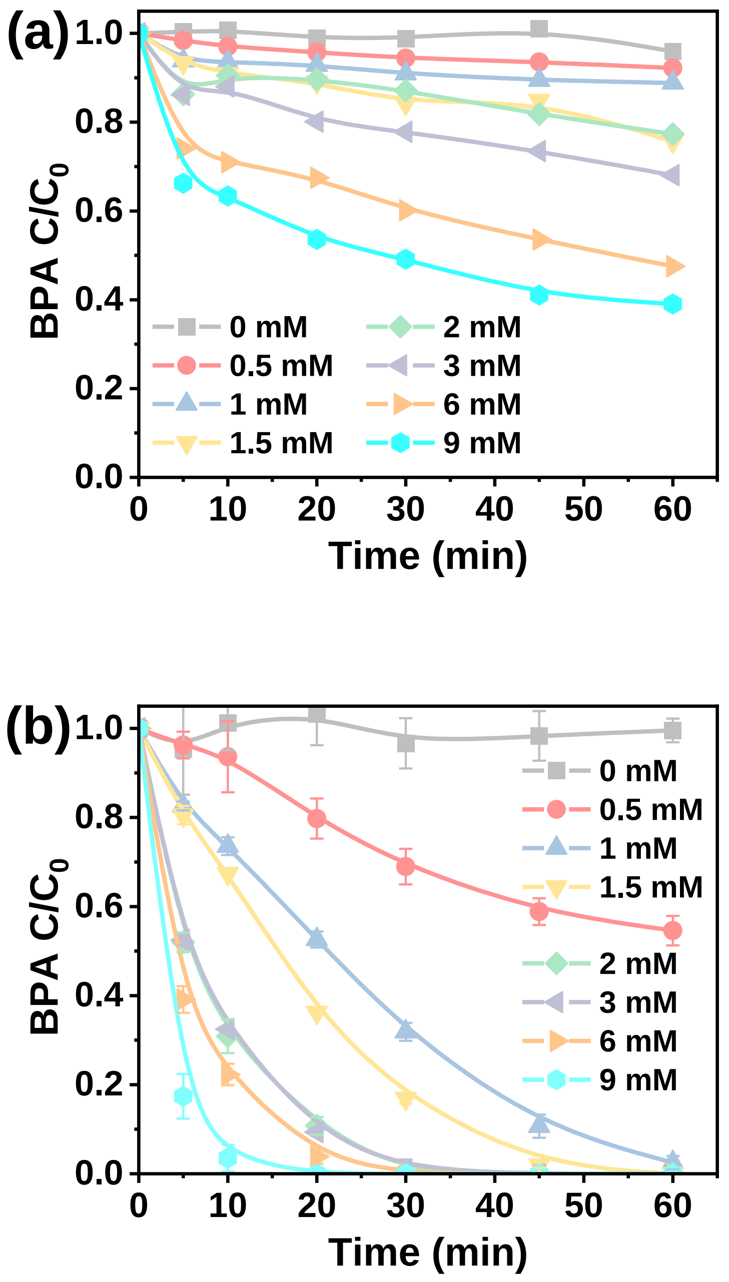

Figure S12. BPA degradation by (a) TQBQ-Cu/PS, (b) TQBQ-Cu/PS/*VL* systems with different concentrations of PS. ([BPA] = 20 ppm, [TQBQ-Cu] = 0.1 g/L with 0.5 mM Cu^{2+} pre-adsorbed)

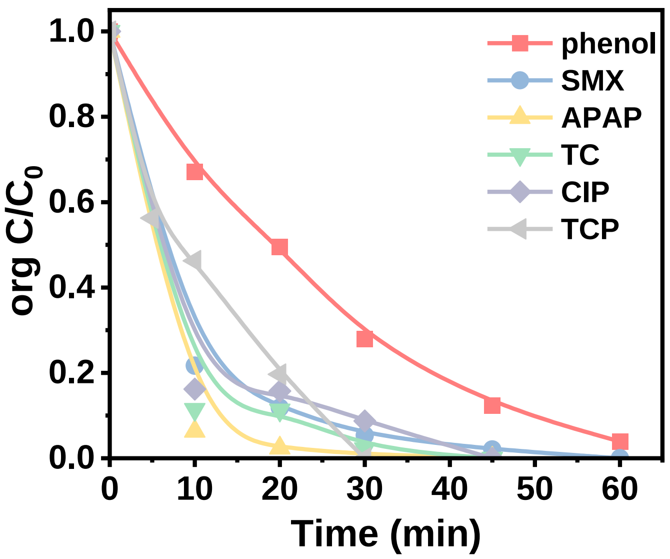

Figure S13. Degradation of different organic compounds by TQBQ-Cu/PS/*VL* system: phenol, sulfamethoxazole (SMX, isoxazole derivative), acetaminophen (APAP, pharmaceutical molecule), tetracycline (TC, antibiotic), ciprofloxacin (CIP, antibiotic), and trichlorophenol (TCP, organochlorine intermediate). ([PS] = 1.5 mM, [TQBQ-Cu] = 0.1 g/L with 0.5 mM Cu^{2+} pre-adsorbed, [PhOH] = 8 ppm, [SMX] = 5 ppm, [APAP] = 5 ppm, [TC] = 5 ppm, [CIP] = 5 ppm, [TCP] = 10 ppm)

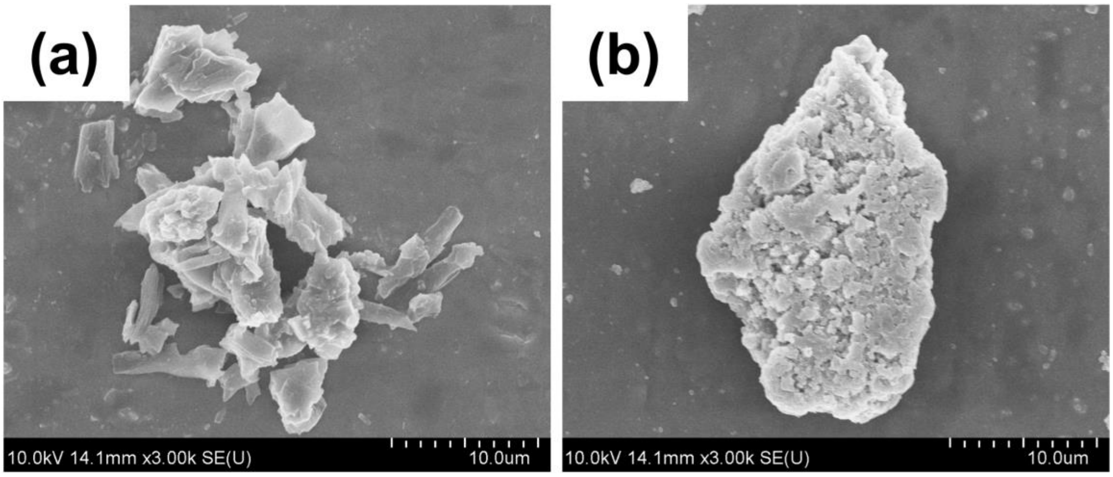

Figure S14. SEM images of (a) TQBQ-COF and (b) TQBQ-Cu.

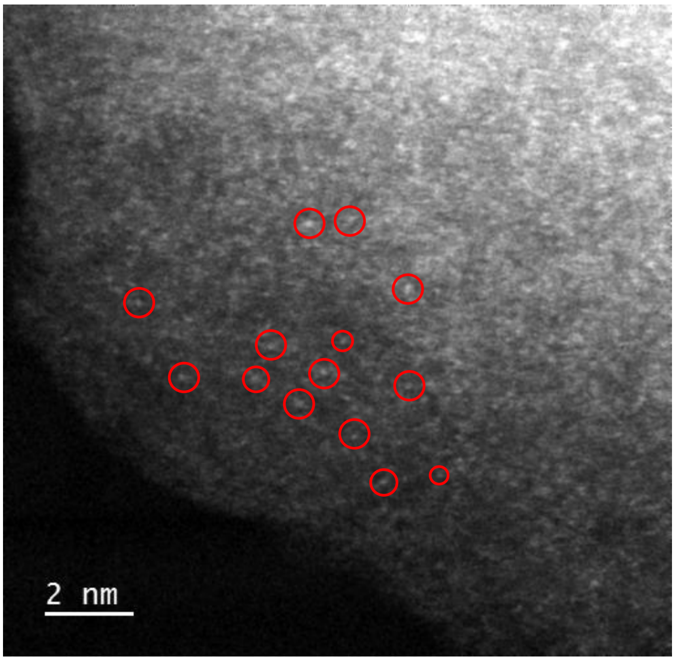

Figure S15. Magnified AC-HAADF-STEM image of TQBQ-Cu.

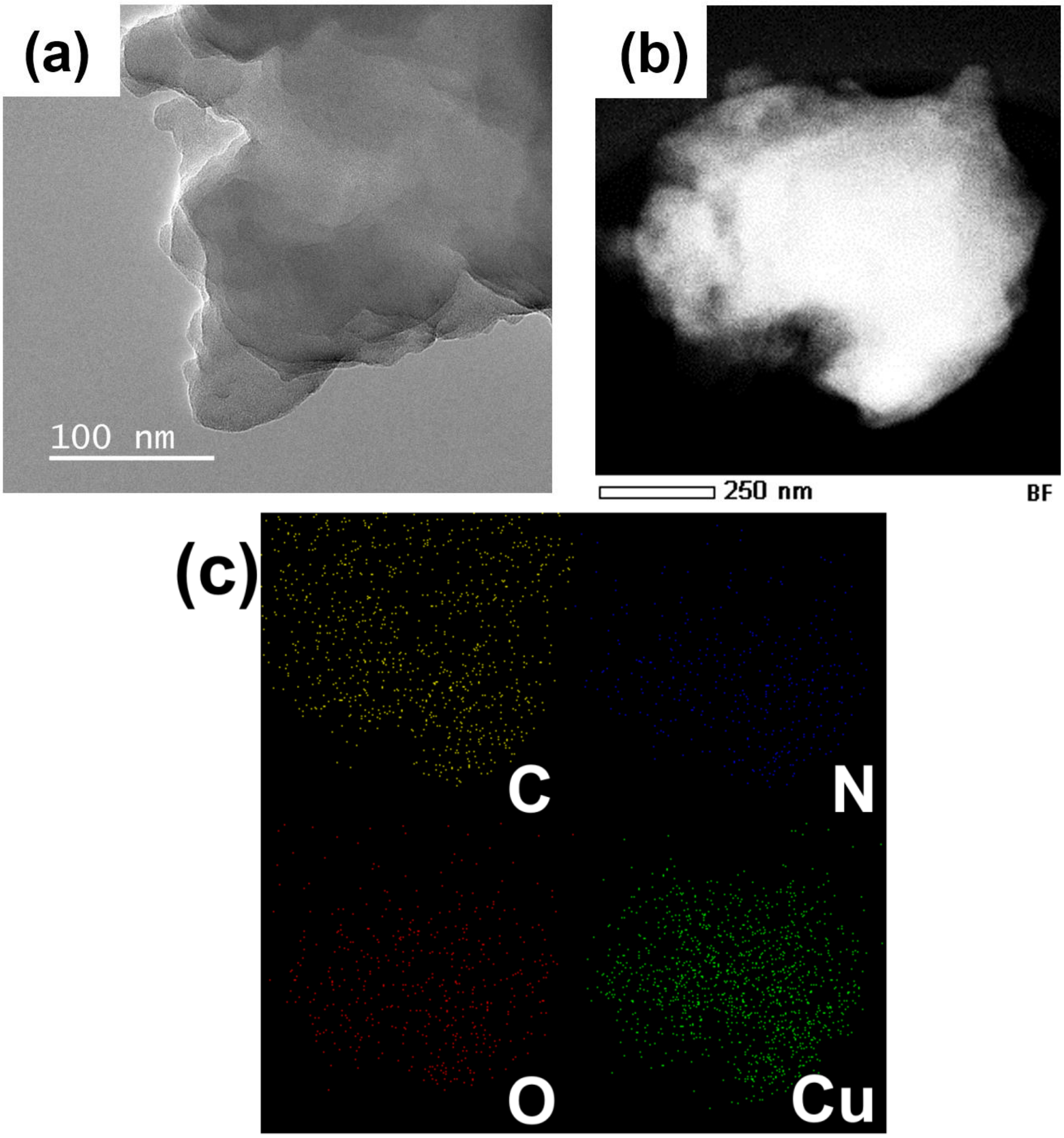

Figure S16. (a) TEM image, (b) HAADF-STEM image and (c) corresponding element mapping images of TQBQ-Cu.

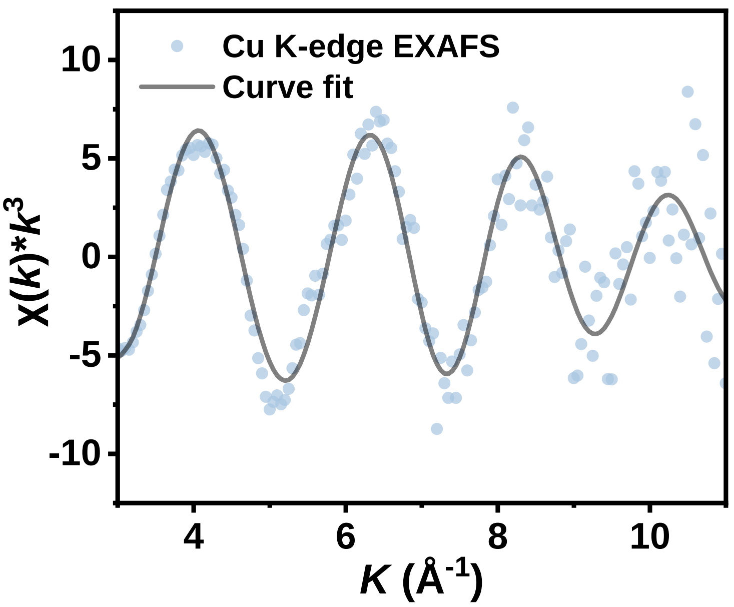

Figure S17. Cu K-edge EXAFS (blue dot) and the curve fit (grey line) for TQBQ-Cu, plotted in k^3-weighted *k*-space.

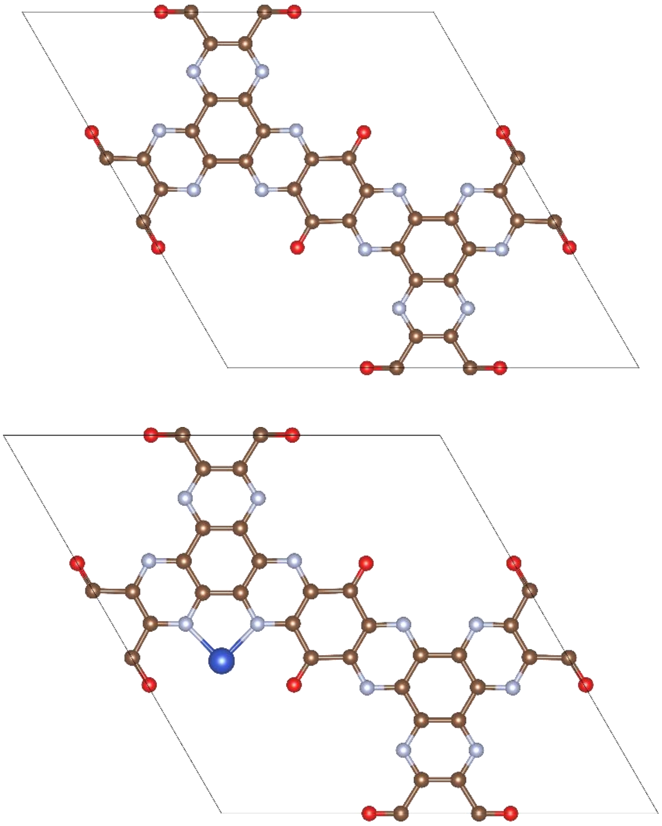

Figure S18. Calculated model of TQBQ-COF and TQBQ-Cu.

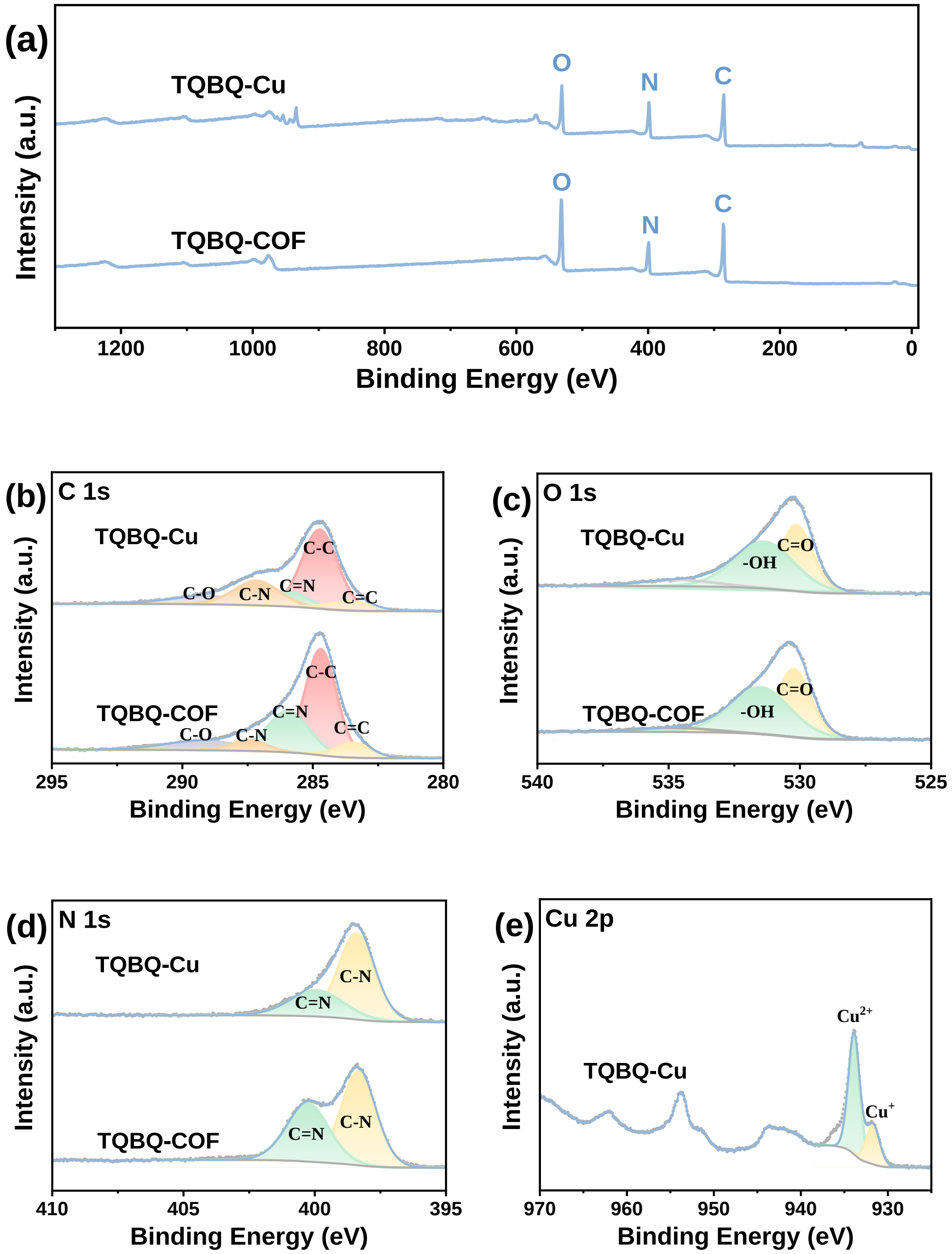

Figure S19. (a) XPS survey spectra of TQBQ-COF and TQBQ-Cu. High-resolution XPS (b) C 1s, (c) O 1s and (d) N 1s spectra of TQBQ-COF and TQBQ-Cu.

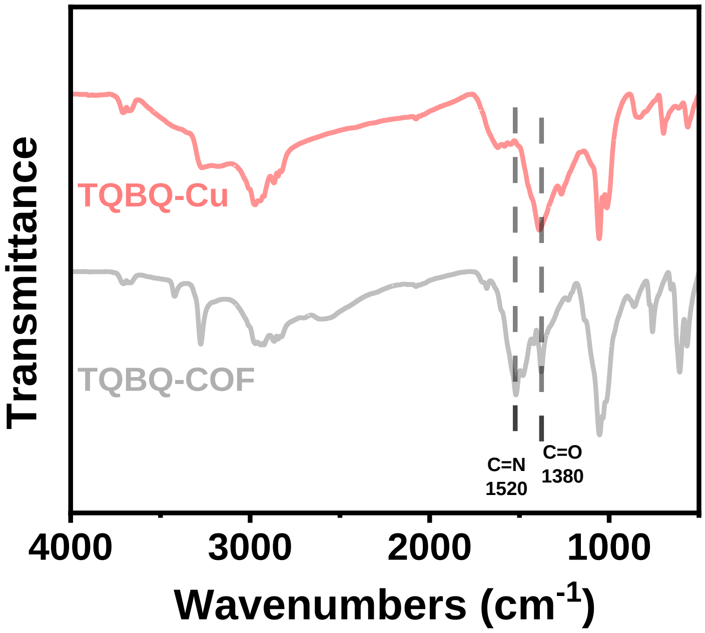

Figure S20. Fourier Transform Infrared (FT-IR) spectra of TQBQ-COF and TQBQ-Cu.

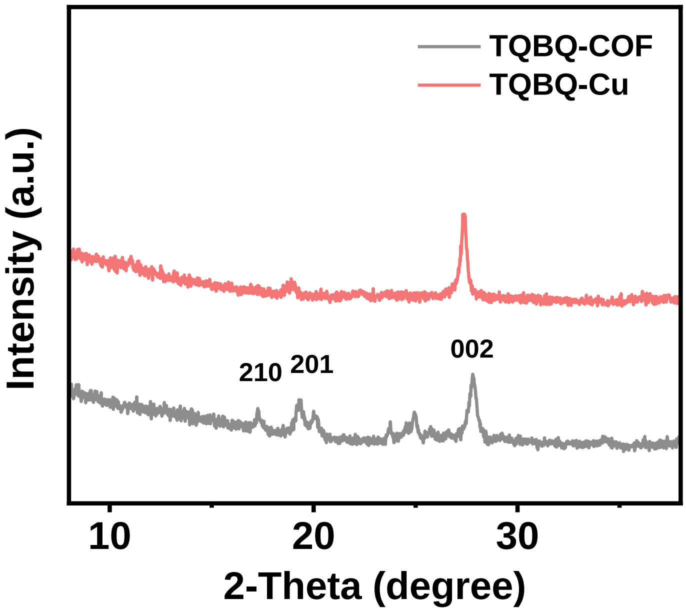

Figure S21. Powder X-ray diffraction patterns of TQBQ-COF and TQBQ-Cu.

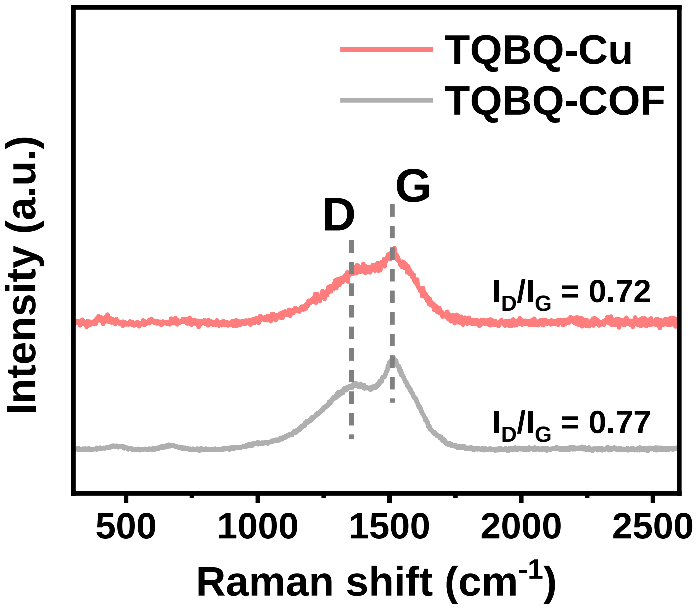

Figure S22. Raman spectra of TQBQ-COF and TQBQ-Cu.

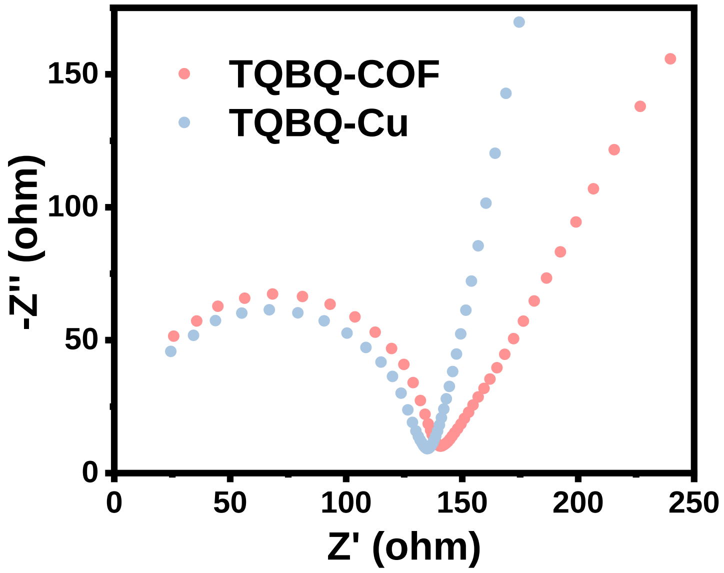

Figure S23. EIS plots of TQBQ-COF and TQBQ-Cu.

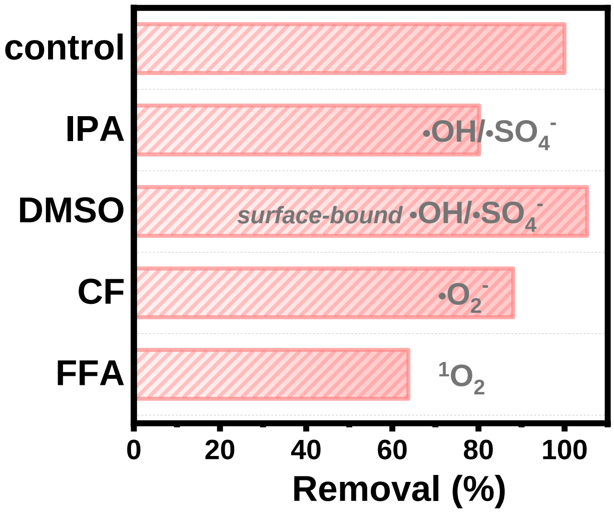

Figure S24. BPA degradation by TQBQ-Cu/PS systems (w/o light) under different quenchers. ([BPA] = 20 ppm, [PS] = 1.5 mM, [Catalyst] = 0.1 g/L, [IPA] = 5 mM, [DMSO] = 10 mM, [CF] = 120 mM, [FFA] = 5 mM)

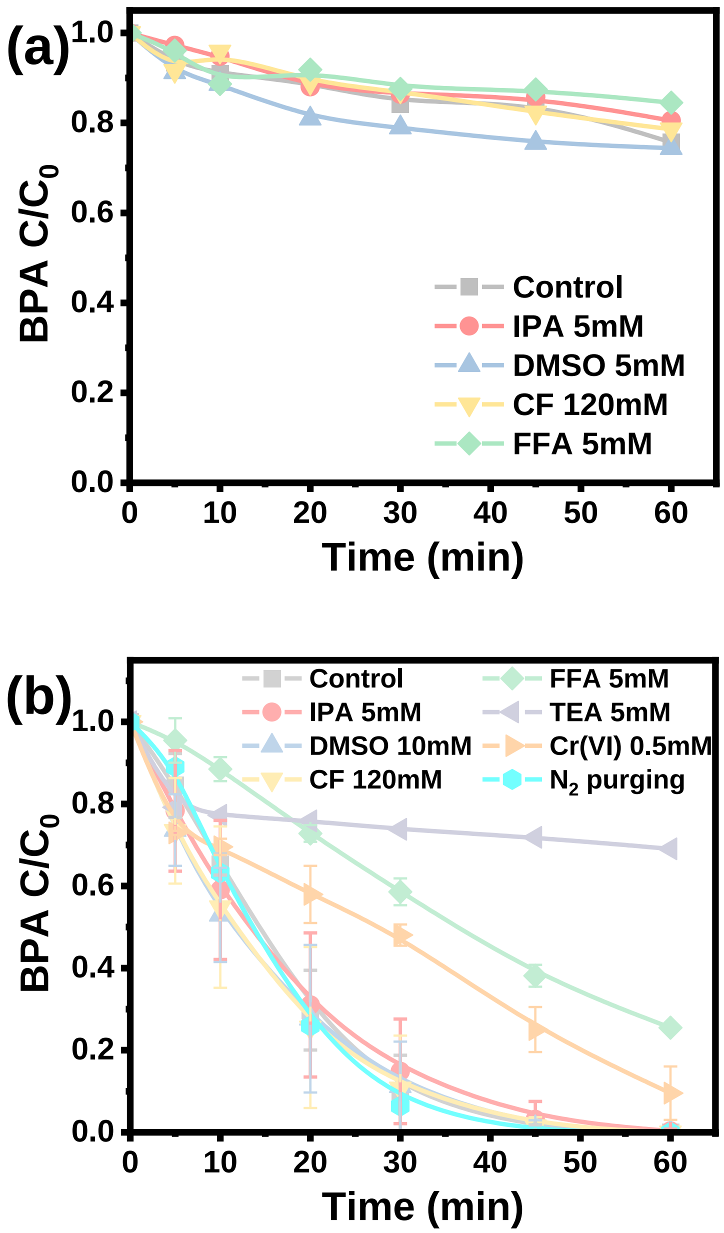

Figure S25. BPA degradation by (a) TQBQ-Cu/PS and (b) TQBQ-Cu/PS/VL systems under different quenchers. ([BPA] = 20 ppm, [PS] = 1.5 mM, [Catalyst] = 0.1 g/L, [IPA] = 5 mM, [DMSO] = 10 mM, [CF] = 120 mM, [FFA] = 5 mM, [TEA] = 5 mM, [Cr (VI)] = 0.5 mM)

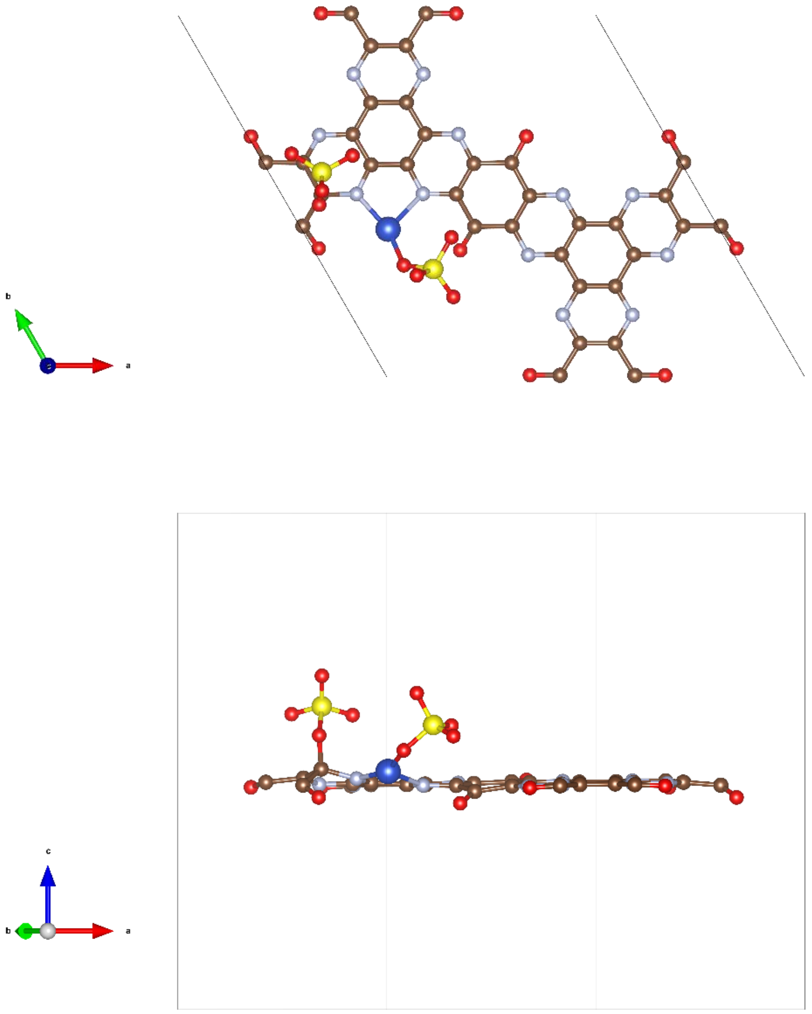

Figure S26. Adsorption of PS by TQBQ-Cu calculated by DFT calculation.

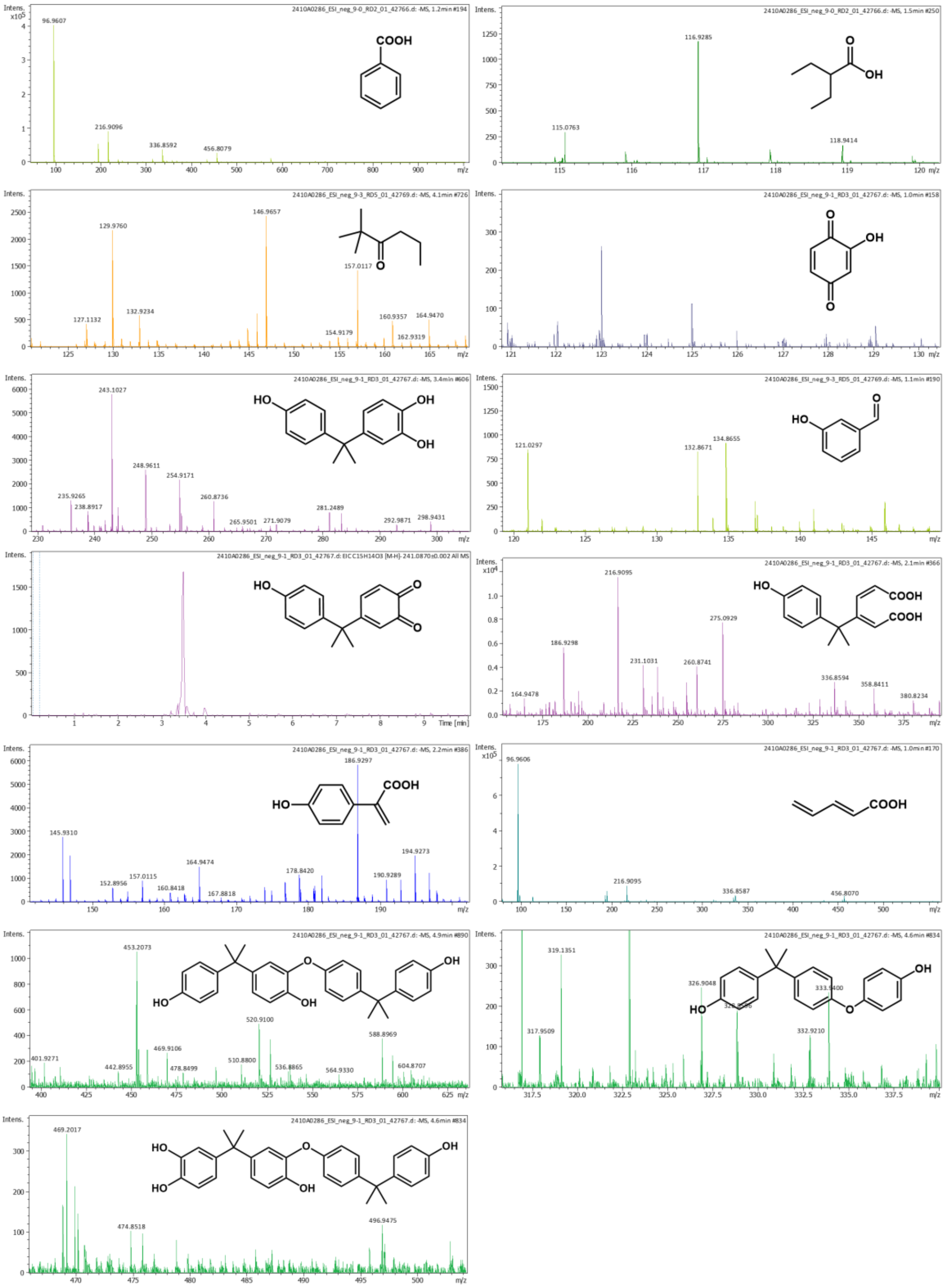

Figure S27. Degradation intermediates and products of BPA.

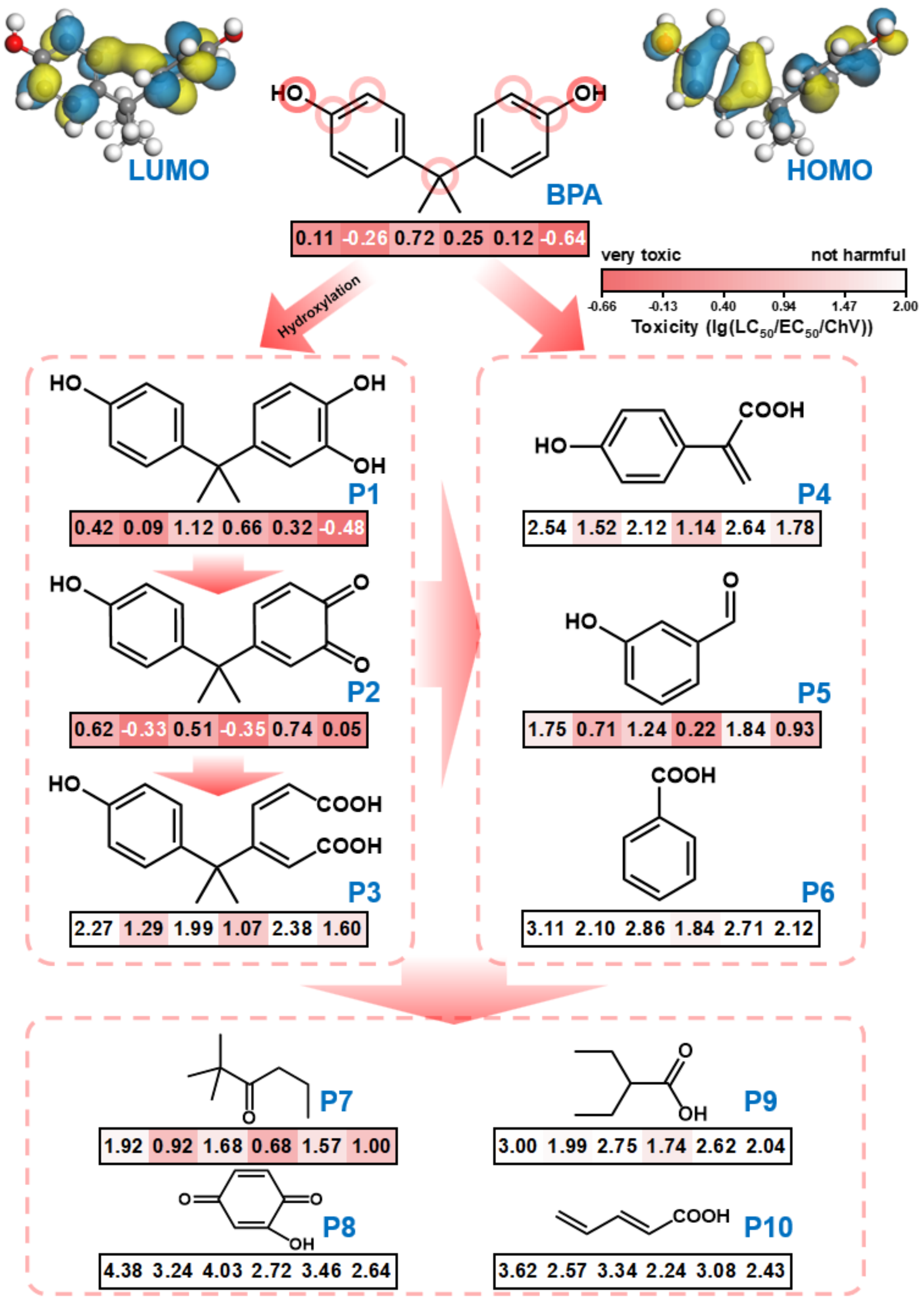

Figure S28. HOMO and LUMO of BPA molecule, degradation pathway analysis of BPA, and corresponding toxicity evaluation of every intermediate provided by ECOSAR. The six indices under every substance represents its estimated Log10 toxicity values of 96-h LC_{50} and chronic ChV for fish, 48-h LC_{50} and ChV for daphnid, 96-h EC_{50} and ChV for green algae, from left to right. The overall heatmap is presented in Fig. S26.

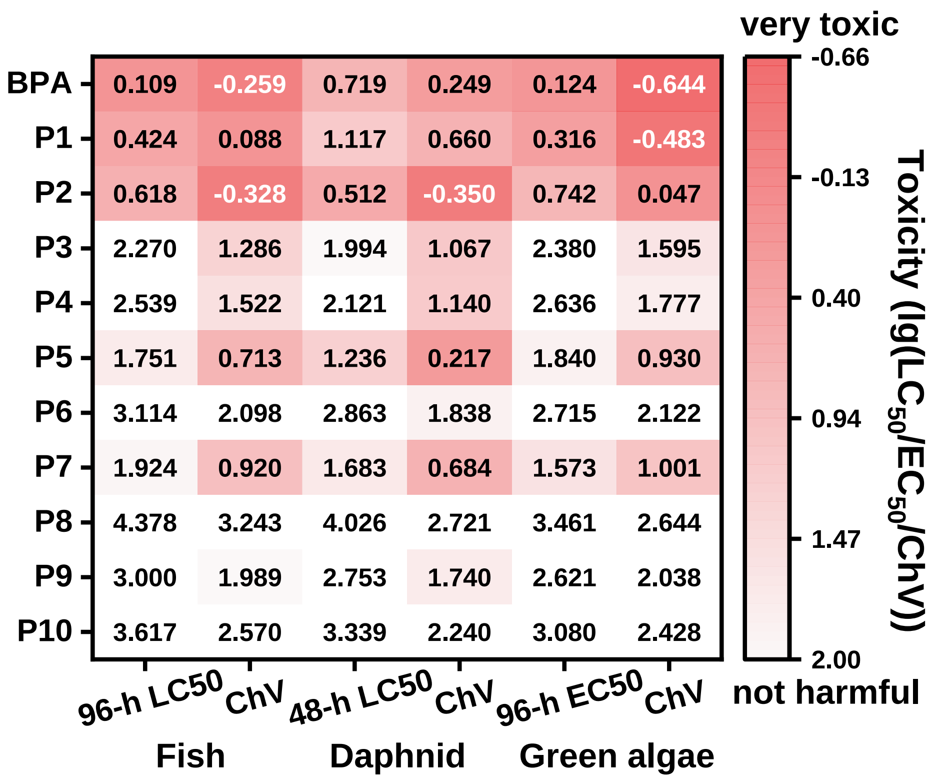

Figure S29. Estimated Log10 values of acute and chronic toxicity for fish, daphnid and green algae of BPA and intermediate products by ECOSAR.

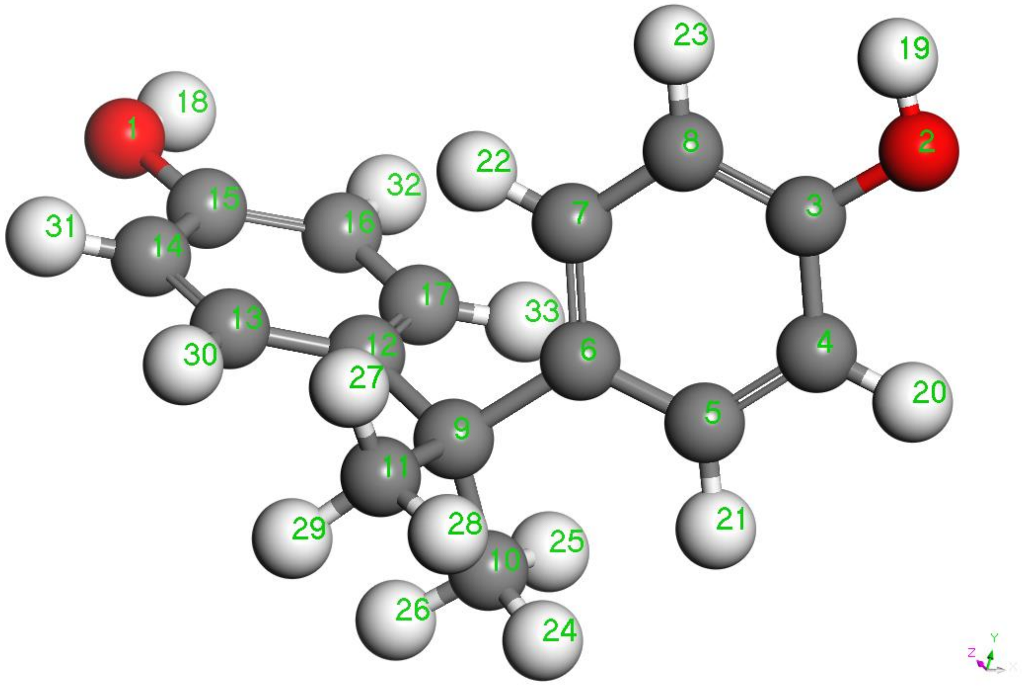

Figure S30. Structure and atom serial numbers of BPA.

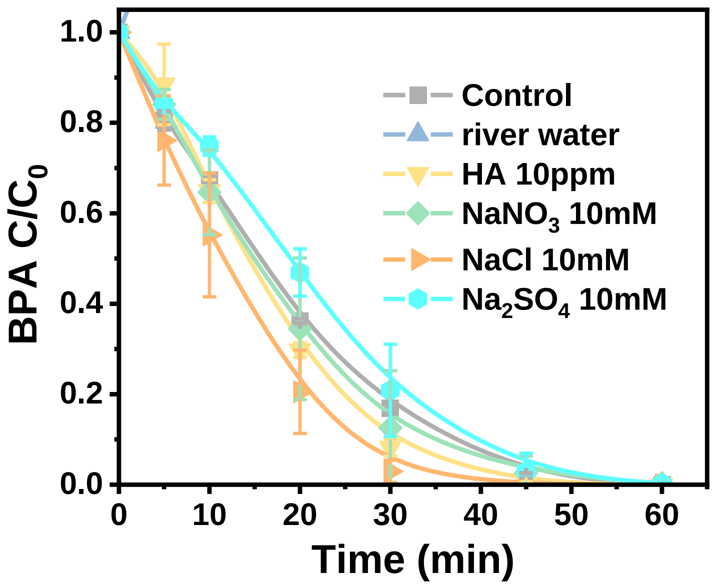

Figure S31. BPA degradation by TQBQ-Cu/PS/*VL* system in the presence of different environmental substances. ([BPA] = 20 ppm, [PS] = 1.5 mM, [Catalyst] = 0.1 g/L. The river water was obtained at Pearl River from the north coast of HEMC, Guangzhou)

Table S1. Efficiency comparison of TQBQ-Cu and other reported photocatalysts for BPA degradation.

Photocatalyst	Catalyst dosage (g/L)	$[BPA]_0$ (mg/L)	$[PMS]_0/[PS]_0$ (g/L)	Lamp power (kW)	BPA removal (C/C_0)	k-value (μmol/g/h)	ref
**Metal-free materials**							
$g\text{-}C_3N_4$	0.5	5	PS 2.2	0.45	1	0.0081	[2]
$g\text{-}C_3N_4$	0.15	9	PMS 1.0	0.35	0.55	0.0407	[2]
$PI\text{-}g\text{-}C_3N_4$	1	10	PMS 3.1	0.3	0.96	0.0117	[2]
GA-CN	1	10	PMS 0.23		1	0.0041	[3]
$PI\text{-}g\text{-}C_3N_4$	1	10	PMS 0.75	0.3	0.92	0.0112	[4]
S doped C_3N_4	0.3	50	PMS 0.07	0.15	0.5	0.0507	[5]
$g\text{-}C_3N_4$ nanosheet	0.5	5	PS 0.23	0.15	1	0.0081	[6]
**Metal@Carbon materials**							
M-CN	1	10	PMS 0.15	0.5	0.97	0.0118	[7]
FeOOH/ $g\text{-}C_3N_4$	1	10	PMS 0.08	0.5	0.93	0.0075	[8]
FONGK-10	1	10	PMS 0.08	0.3	1	0.0182	[9]
Fe^{3+}/ $g\text{-}C_3N_4$	0.5	0.13	PMS 0.015	/	1	0.0002	[10]
$Fe\text{-}UIO66\text{-}NH_2$	0.33	20	PMS 0.33	/	0.98	0.0719	[11]
**Metal-based materials**							
$Fe_2(MoO_4)_3$	1	10	PS 1.1	0.3	1	0.0122	[2]
$WO_3@MoS_2/Ag$	0.8	10	PMS 1.0	0.5	0.93	0.0062	[2]
**COFs**							
COF-PRD	0.3	10	PMS 0.5	0.3	0.98	0.0159	[2]
$CTF\text{-}SD_2$	0.3	23	PMS 0.3	0.3	1	0.0389	[12]
DAQ-COF	0.25	20	PMS 0.23	0.01	1	0.0973	[13]
**Metal-COFs**							
$MIL101\text{-}NH_2@TpMA$	0.25	50	PS 0.5	0.5	0.9	0.0548	[14]
$UIO66\text{-}NH_2@TpMA$	0.25	50	PS 0.5	0.5	0.7	0.0426	[14]

TQBQ-Cu (0.5mM Cu)	0.1	20	PS 0.29	0.01	1	0.243	This
TQBQ-Cu (0.5mM Cu)	0.1	20	PS 1.73	0.01	1	0.973	work

The degradation efficiency *k* is defined as the change of pollutant concentration per hour per catalyst concentration [15]:

$$k = [\text{pollutant removal}]/[\text{time}]/[\text{catalyst}]\ (h^{-1})$$

Table S2. Second-order rate constant (*k*) for the common quenchers with corresponding ROSs.

Quenchers	$^1O_2$ (M^{-1} s^{-1})	•OH (M^{-1} s^{-1})	SO4•- (M^{-1} s^{-1})	$•O_2^-$ (M^{-1} s^{-1})
Furfuryl alcohol (FFA)	9.7×10^8 [16]	1.5×10^{10} [16]	~0 [17]	
L-histidine	3.2×10^7 [18]	5.0×10^9 [19]	2.5×10^9 [19]	
β-carotene	$(2\text{-}3) \times 10^{10}$ [20]			2.5×10^8 [20]
NaN_3	1×10^9 [21]	1.2×10^{10} [21]	2.52×10^9 [21]	
Methanol	negligible[21]	9.7×10^8 [22]	$(1.6\text{-}7.7) \times 10^7$ [18]	
Ethanol	negligible[21]	$(1.2\text{-}2.8) \times 10^9$ [16]	$(1.6\text{-}7.7) \times 10^7$ [16]	
Iso-propanol (IPA)	negligible[23]	2.8×10^9 [18]	8.3×10^7 [18]	
Tert-butanol	negligible[21]	$(3.8\text{-}7.6) \times 10^8$ [16]	$(4.0\text{-}9.1) \times 10^5$ [16]	
Nitrobenzene		4.7×10^9 [24]	$< 10^6$ [24]	
Benzoic acid		2.1×10^9 [24]	1.2×10^9 [24]	
p-benzoquinone				$(0.9\text{-}1) \times 10^9$ [22]
Chloroform (CF)				3×10^{10} [18]

As reported by Rodgers *et al.* [23], $k_{H2O} = 4300\ M^{-1}s^{-1}$, while k of those alcohols above are around $4 \times 10^3\ M^{-1}s^{-1}$. Therefore, in water treatment systems, the inhibition of $^1O_2$ by alcohols is negligible.

Table S3. Identified intermediates during BPA degradation based on LC-Q-TOF MS spectra in SI Fig. S24.

Products	m/z	Molecular weight	Molecular formula	Proposed structure
BPA	227.11	228.29	C15H16O2	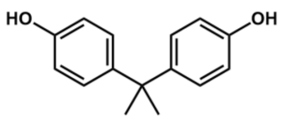
P1	243.1027	244.29	C15H16O3	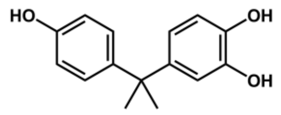
P2	241.0870	242.27	C15H14O3	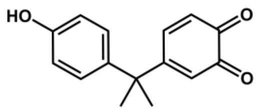
P3	275.0925	276.29	C15H16O5	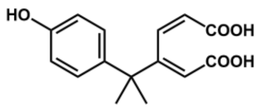
P4	163.0401	164.16	C9H8O3	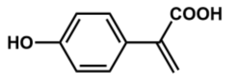
P5	121.0295	122.12	C7H6O2	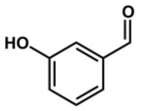
P6	121.0295	122.12	C7H6O2	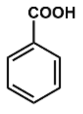
P7	127.1128	128.22	C8H16O	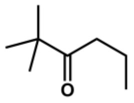
P8	123.0088	124.10	C6H4O3	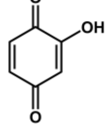

P9	115.0765	116.16	C6H12O2	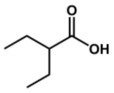
P10	97.0295	98.10	C5H6O2	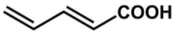
P11	453.2071	454.57	C30H30O4	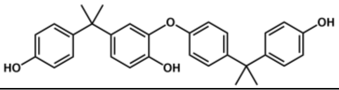
P12	469.2020	470.57	C30H30O5	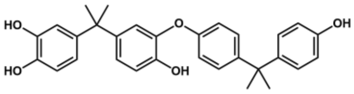
P13	319.1340	320.39	C21H20O3	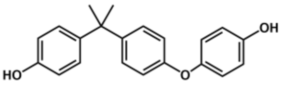

Table S4. Calculated Fukui index of BPA.

Atom	f^-	f^+	f^0
1 O	0.0724	0.0291	0.0508
2 O	0.0119	0.0083	0.0101
3 C	0.0119	0.0083	0.0101
4 C	0.0483	0.0122	0.0303
5 C	0.0335	0.0604	0.047
6 C	0.0513	0.0649	0.0581
7 C	0.0541	0.0306	0.0423
8 C	0.0369	0.0665	0.0517
9 C	0.0271	0.0503	0.0387
10 C	0.0724	0.0291	0.0508
11 C	0.0541	0.0306	0.0423
12 C	0.0368	0.0665	0.0517
13 C	0.0271	0.0503	0.0387
14 C	0.0483	0.0122	0.0303
15 C	0.0335	0.0604	0.047
16 C	0.0513	0.0649	0.0581
17 C	0.0043	-0.0006	0.0019

The atomic number was presented in SI Fig. S30.

Table S5. Properties of the copper electroplating effluent.

$[Cu]_0$	COD	pH
67.42 mM	24080 mg/L	2.0

The real wastewater sample was used for photo-Fenton process without further adjustment.

Table S6. Cu removal of treated copper electroplating effluent at different reaction interval after 0.1M NaOH addition.

Time interval	0h	0h (after COF addition)	2h
Aqueous Cu (mM)	67.4	58.2	57.1
Residual Cu after 0.1M NaOH addition (mM)	67.4	58.2	21.1
Removal (%)	0	0	63.0

Table S7. Curve-fit parameters for Cu K-edge EXAFS for TQBQ-Cu.

Path	d^b/ Å	N	R / Å	σ^2 / Å	ΔE_0 / eV
Cu-N	1.98910	2.1	1.97(1)	0.006(1)	-2.57±1.35

The Cu-N distance d^b was obtained from cif file from DFT calculation. N is the coordination number; R is the fitted distance between Cu and surrounding N coordination atoms; σ^2 is Debye-Waller factor to account for both thermal and structural disorders; ΔE_0 is inner potential correction. Data ranges: $3 \leqslant k \leqslant 10.918$ Å, $1.0 \leqslant R \leqslant 3.0$ Å. The number of variable parameters is 4, out of a total of 9.8 independent data points. R factor is 1.5%.

References

[1] M. Wu, Y. Zhao, B. Sun, Z. Sun, C. Li, Y. Han, L. Xu, Z. Ge, Y. Ren, M. Zhang, Q. Zhang, Y. Lu, W. Wang, Y. Ma, Y. Chen, A 2D covalent organic framework as a high-performance cathode material for lithium-ion batteries, Nano Energy, 70 (2020) 104498. https://doi.org/10.1016/j.nanoen.2020.104498.

[2] F. Liu, Q. Dong, C. Nie, Z. Li, B. Zhang, P. Han, W. Yang, M. Tong, Peroxymonosulfate enhanced photocatalytic degradation of serial bisphenols by metal-free covalent organic frameworks under visible light irradiation: mechanisms, degradation pathway and DFT calculation, Chem. Eng. J., 430 (2022) 132833. https://doi.org/10.1016/j.cej.2021.132833.

[3] C. Tong, L. Jing, M. Xie, M. He, Y. Liu, J. Yuan, Y. Song, Y. Xu, C–O band structure modified broad spectral response carbon nitride with enhanced electron density in photocatalytic peroxymonosulfate activation for bisphenol pollutants removal, J. Hazard. Mater., 432 (2022) 128663. https://doi.org/10.1016/j.jhazmat.2022.128663.

[4] J. Zhang, X. Zhao, Y. Wang, Y. Gong, D. Cao, M. Qiao, Peroxymonosulfate-enhanced visible light photocatalytic degradation of bisphenol A by perylene imide-modified g-C3N4, Appl. Catal., B, 237 (2018) 976-985. https://doi.org/10.1016/j.apcatb.2018.06.049.

[5] K.-Y.A. Lin, Z.-Y. Zhang, Degradation of Bisphenol A using peroxymonosulfate activated by one-step prepared sulfur-doped carbon nitride as a metal-free heterogeneous catalyst, Chem. Eng. J., 313 (2017) 1320-1327. https://doi.org/10.1016/j.cej.2016.11.025.

[6] B. Liu, M. Qiao, Y. Wang, L. Wang, Y. Gong, T. Guo, X. Zhao, Persulfate enhanced photocatalytic degradation of bisphenol A by g-C3N4 nanosheets under visible light irradiation, Chemosphere, 189 (2017) 115-122. https://doi.org/10.1016/j.chemosphere.2017.08.169.

[7] Q. Si, W. Guo, B. Liu, H. Wang, S. Zheng, Q. Zhao, H. Luo, N. Ren, T. Yu, Spin-states-assistance peroxymonosulfate absorption via Mn doped catalyst with/without light for BPA oxidation: The negative contribution of electrons transfer by light, Chem. Eng. J., 443 (2022) 136399. https://doi.org/10.1016/j.cej.2022.136399.

[8] X. Zhang, Y. Liu, C. Li, L. Tian, F. Yuan, S. Zheng, Z. Sun, Fast and lasting electron transfer between γ-FeOOH and g-C3N4/kaolinite containing N vacancies for enhanced visible-light-assisted peroxymonosulfate activation, Chem. Eng. J., 429 (2022) 132374. https://doi.org/10.1016/j.cej.2021.132374.

[9] X. Zhang, S. Yang, C. Li, J. Liang, X. Wang, S. Zheng, Z. Sun, Protrudent electron transfer channels on kaolinite modified iron oxide QDs/N vacancy graphitic carbon nitride driving superior catalytic oxidation, J. Hazard. Mater., 436 (2022)

129244. https://doi.org/10.1016/j.jhazmat.2022.129244.

[10] L. Xu, L. Qi, Y. Han, W. Lu, J. Han, W. Qiao, X. Mei, Y. Pan, K. Song, C. Ling, L. Gan, Improvement of Fe2+/peroxymonosulfate oxidation of organic pollutants by promoting Fe2+ regeneration with visible light driven g-C3N4 photocatalysis, Chem. Eng. J., 430 (2022) 132828. https://doi.org/10.1016/j.cej.2021.132828.

[11] S.-W. Lv, X. Wang, X. Wei, Y. Zhang, Y. Cong, L. Che, Introduction of cluster-to-metal charge transfer in UiO-66-NH2 for enhancing photocatalytic degradation of bisphenol a in the existence of peroxymonosulfate, Sep. Purif. Technol., 292 (2022) 121018. https://doi.org/10.1016/j.seppur.2022.121018.

[12] T. Zeng, S. Jin, S. Li, J. Bao, Z. Jin, D. Wang, F. Dong, H. Zhang, S. Song, Covalent Triazine Frameworks with Defective Accumulation Sites: Exceptionally Modulated Electronic Structure for Solar-Driven Oxidative Activation of Peroxymonosulfate, Environ. Sci. Technol., 56 (2022) 9474-9485. https://doi.org/10.1021/acs.est.2c00126.

[13] Y. Tao, Y. Hou, H. Yang, Z. Gong, J. Yu, H. Zhong, Q. Fu, J. Wang, F. Zhu, G. Ouyang, Interlayer synergistic reaction of radical precursors for ultraefficient 1O2 generation via quinone-based covalent organic framework, Proc. Natl. Acad. Sci. U.S.A, 121 (2024) e2401175121. https://doi.org/10.1073/pnas.2401175121.

[14] S.-W. Lv, J.-M. Liu, C.-Y. Li, N. Zhao, Z.-H. Wang, S. Wang, Two novel MOFs@COFs hybrid-based photocatalytic platforms coupling with sulfate radical-involved advanced oxidation processes for enhanced degradation of bisphenol A, Chemosphere, 243 (2020) 125378. https://doi.org/10.1016/j.chemosphere.2019.125378.

[15] Y. Chen, G. Zhang, H. Liu, J. Qu, Confining Free Radicals in Close Vicinity to Contaminants Enables Ultrafast Fenton-like Processes in the Interspacing of MoS2 Membranes, Angew. Chem. Int. Ed., 58 (2019) 8134-8138. https://doi.org/10.1002/anie.201903531.

[16] J. Liang, L. Fu, Activation of peroxymonosulfate (PMS) by Co3O4 quantum dots decorated hierarchical C@Co3O4 for degradation of organic pollutants: Kinetics and radical-nonradical cooperation mechanisms, Appl. Surf. Sci., 563 (2021) 150335. https://doi.org/10.1016/j.apsusc.2021.150335.

[17] X. Zhang, J. Yao, Z. Zhao, J. Liu, Degradation of haloacetonitriles with UV/peroxymonosulfate process: Degradation pathway and the role of hydroxyl radicals, Chem. Eng. J., 364 (2019) 1-10. https://doi.org/10.1016/j.cej.2019.01.029.

[18] L. Wang, X. Lan, W. Peng, Z. Wang, Uncertainty and misinterpretation over identification, quantification and transformation of reactive species generated in catalytic oxidation processes: A review, J. Hazard. Mater., 408 (2021) 124436. https://doi.org/10.1016/j.jhazmat.2020.124436.

[19] L. Wu, Q. Lin, H. Fu, H. Luo, Q. Zhong, J. Li, Y. Chen, Role of sulfide-modified nanoscale zero-valent iron on carbon nanotubes in nonradical activation of peroxydisulfate, J. Hazard. Mater., 422 (2022) 126949.

https://doi.org/10.1016/j.jhazmat.2021.126949.

[20] S.Y. Wang, H. Jiao, Scavenging Capacity of Berry Crops on Superoxide Radicals, Hydrogen Peroxide, Hydroxyl Radicals, and Singlet Oxygen, Journal of Agricultural and Food Chemistry, 48 (2000) 5677-5684. https://doi.org/10.1021/jf000766i.

[21] Y. Zhou, J. Jiang, Y. Gao, J. Ma, S.Y. Pang, J. Li, X.T. Lu, L.P. Yuan, Activation of Peroxymonosulfate by Benzoquinone: A Novel Nonradical Oxidation Process, Environ. Sci. Technol., 49 (2015) 12941-12950. https://doi.org/10.1021/acs.est.5b03595.

[22] X. Li, J. Wang, X. Duan, Y. Li, X. Fan, G. Zhang, F. Zhang, W. Peng, Fine-Tuning Radical/Nonradical Pathways on Graphene by Porous Engineering and Doping Strategies, ACS Catal., 11 (2021) 4848-4861. https://doi.org/10.1021/acscatal.0c05089.

[23] M.A.J. Rodgers, Solvent-induced deactivation of singlet oxygen: additivity relationships in nonaromatic solvents, J. Am. Chem. Soc., 105 (1983) 6201-6205. https://doi.org/10.1021/ja00358a001.

[24] J. Dou, J. Cheng, Z. Lu, Z. Tian, J. Xu, Y. He, Biochar co-doped with nitrogen and boron switching the free radical based peroxydisulfate activation into the electron-transfer dominated nonradical process, Appl. Catal., B, 301 (2022) 120832. https://doi.org/10.1016/j.apcatb.2021.120832.
